# Association between systemic inflammatory indicators with the survival of chronic kidney disease: a prospective study based on NHANES

**DOI:** 10.3389/fimmu.2024.1365591

**Published:** 2024-04-08

**Authors:** Yuan Chen, Yanfang Nie, Jiaying Wu, Chunsheng Li, Lu Zheng, Bixiu Zhu, Yu Min, Tao Ling, Xiaozhu Liu

**Affiliations:** ^1^Department of Nephrology, Taizhou Central Hospital (Taizhou University Hospital), Taizhou, Zhejiang, China; ^2^Department of Biotherapy and National Clinical Research Center for Geriatrics, Cancer Center, West China Hospital, Sichuan University, Sichuan, China; ^3^Department of Pharmacy, Suqian First Hospital, Suqian, China; ^4^Department of Critical Care Medicine, Beijing Shijitan Hospital, Capital Medical University, Beijing, China

**Keywords:** chronic kidney disease, systemic inflammatory index, all-cause mortality, NHANES, prospective study

## Abstract

**Background:**

systemic inflammation disorders were observed in chronic kidney disease (CKD). Whether the systemic inflammatory indicators could be optimal predictors for the survival of CKD remains less studied.

**Methods:**

In this study, participants were selected from the datasets of the National Health and Nutrition Examination Survey (NHANES) between 1999 to 2018 years. Four systemic inflammatory indicators were evaluated by the peripheral blood tests including systemic immune-inflammation index (SII, platelet*neutrophil/lymphocyte), neutrophil-to-lymphocyte ratio (NLR), platelet-to-lymphocyte ratio (PLR), lymphocyte-to-monocyte ratio (LMR). Kaplan-Meier curves, restricted cubic spline (RCS), and Cox regression analysis were used to evaluate the association between the inflammatory index with the all-cause mortality of CKD. Receiver operating characteristic (ROC) and concordance index (C-index) were used to determine the predictive accuracy of varied systemic inflammatory indicators. Sensitive analyses were conducted to validate the robustness of the main findings.

**Results:**

A total of 6,880 participants were included in this study. The mean age was 67.03 years old. Among the study population, the mean levels of systemic inflammatory indicators were 588.35 in SII, 2.45 in NLR, 133.85 in PLR, and 3.76 in LMR, respectively. The systemic inflammatory indicators of SII, NLR, and PLR were all significantly positively associated with the all-cause mortality of CKD patients, whereas the high value of LMR played a protectable role in CKD patients. NLR and LMR were the leading predictors in the survival of CKD patients [Hazard ratio (HR) =1.21, 95% confidence interval (CI): 1.07-1.36, p = 0.003 (3^rd^ quartile), HR = 1.52, 95%CI: 1.35-1.72, p<0.001 (4^th^ quartile) in NLR, and HR = 0.83, 95%CI: 0.75-0.92, p<0.001 (2^nd^ quartile), HR = 0.73, 95%CI: 0.65-0.82, p<0.001 (3^rd^ quartile), and = 0.74, 95%CI: 0.65-0.83, p<0.001 (4^th^ quartile) in LMR], with a C-index of 0.612 and 0.624, respectively. The RCS curves showed non-linearity between systemic inflammatory indicators and all-cause mortality risk of the CKD population.

**Conclusion:**

Our study highlights that systemic inflammatory indicators are important for predicting the survival of the U.S. population with CKD. The systemic inflammatory indicators would add additional clinical value to the health care of the CKD population.

## Introduction

Currently, chronic kidney disease (CKD) is an important contributor to morbidity, impaired health-related quality of life (HRQOL), and premature death from noncommunicable diseases, is defined as a reduced glomerular filtration rate (GFR), increased urinary albumin excretion, or both, and is a major public health issue (1–3). In 2017, it was estimated that the prevalence of CKD was estimated as 9.1% with approximately 700 million cases in the world’s population (1). In the United States, half of the population is projected to develop the disease throughout their lifetime, and more than 30 million people already have CKD (4). Population with CKD are at remarkably increased risk of cardiovascular disease, and mortality compared to the general population (4). Notably, CKD resulted in 1.2 million deaths and attributed to an additional 1.4 million deaths from cardiovascular disease (1). Therefore, epidemiological studies are warranted to determine new biomarkers for high-risk CKD subpopulation, which would help nephrologists to make timely clinical management decisions.

Systemic inflammatory disorders were frequently observed in CKD patients (5–7). The pro-inflammation condition contributes to the deterioration of kidney function (8–10). The epidemiological and genetic associations between serum levels of systemic inflammation with the incidence and progress of CKD have been established (7, 11–13). Historically, prognostic factors determined in cardiovascular disease (CVD) were prevalent among patients with CKD. However, it might not fully reflect the increased mortality rates among the CKD population. Notably, recent review literature suggested that even low-grade inflammation would play a decisive role in the all-cause mortality of these patients (14). For this reason, identifying the representative but simple systemic inflammatory indicators for predicting survival among CKD patients would bring considerable cost-benefits in clinical practice (4, 14, 15). Of note, several composite inflammatory indicators, including but not limited to systemic immune-inflammation index (SII), neutrophil-to-lymphocyte ratio (NLR), platelet-to-lymphocyte ratio (PLR), and lymphocyte-to-monocyte ratio (LMR), have been validated to be feasible in predicting the prognosis of various cancers and inflammatory diseases (16–19). These composite indicators, comprising biomarkers easily available in clinical settings such as peripheral lymphocytes, platelets, neutrophils, and monocytes, offer a comprehensive reflection of both local immune status and systemic inflammation condition (20–23). Whether these newly developed indicators presented equivalent predictive value in CKD has not been fully investigated. To date, the available evidence regarding this issue was mainly conducted with single center experience or especially interest in the single inflammatory indicator (24, 25). Moreover, whether the conclusions could be generalized to other countries or regions remains unclear.

To fill the mentioned research gaps, we aim to conduct a prospective study to evaluate the association between varied systemic inflammatory indicators with all-cause mortality among the CKD population in the U.S., based on a large-scale, population-based cohort. Besides, we also compared the predictive value of each inflammatory index.

## Materials and methods

### Data source

The National Health and Nutrition Examination Survey (NHANES) is a representative, ongoing, repeated series of epidemiological surveys regarding the health and nutritional conditions of the noninstitutionalized civilian population in the U.S. The survey contains a wide range of indicators of health and well-being by utilizing a combination of self-reported records and objective physical examinations. Detailed information on NHANES can be found elsewhere (https://www.cdc.gov/nchs/nhanes/). The National Center for Health Statistics Ethics Review Board has approved the NHANES study because the data from NHANES is anonymous and publicly available. All participants provided informed consent. We reported this study following the reporting of observational studies in epidemiology (STROBE) criteria (26).

### Study population

**Figure 1:**
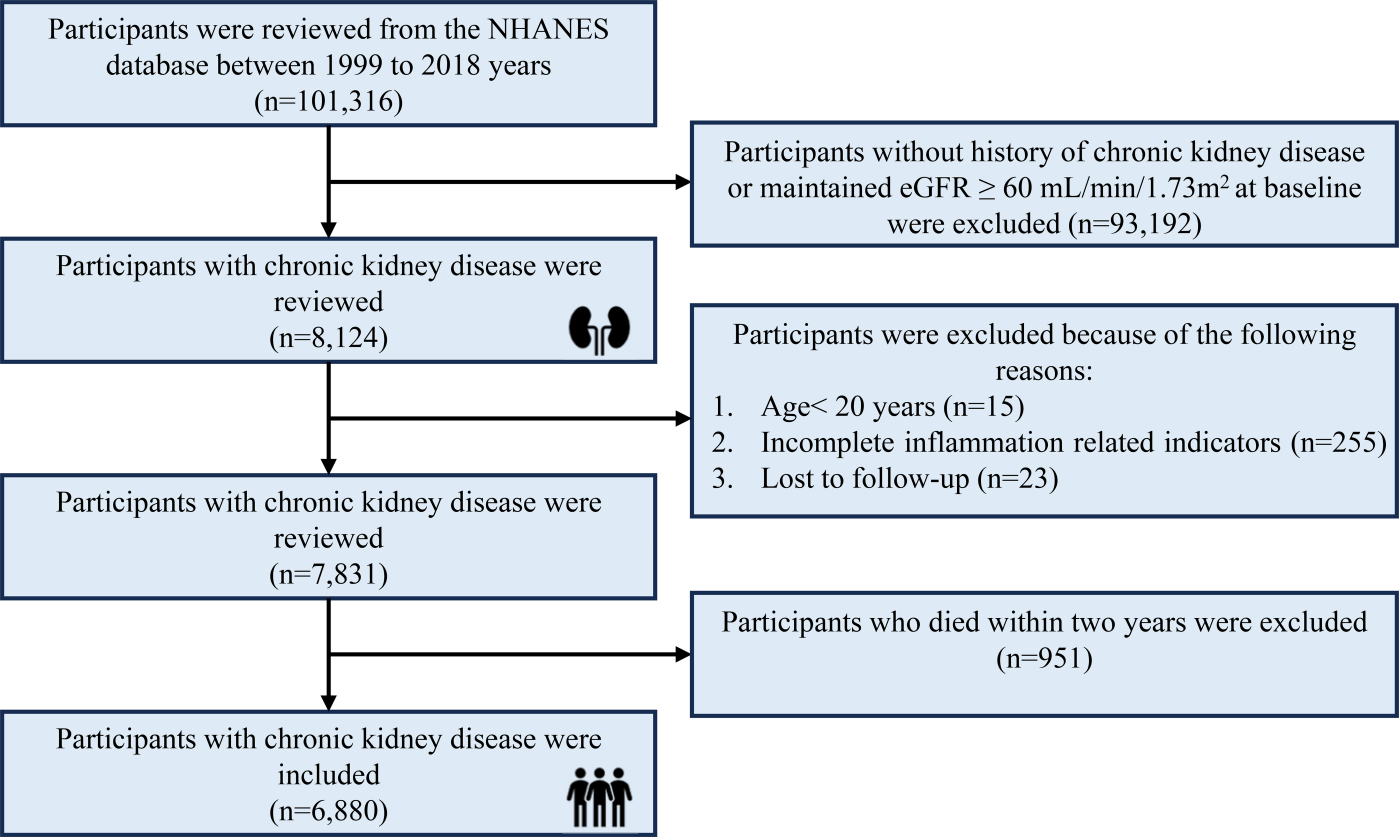
The selection process of participants in this study.

Adult participants were selected from the NHANES database within ten cycles of the surveys (1999–2000 to 2017–2018). Participants with a history of CKD were included in the present study. To evaluate the association between inflammatory indicators with the survival of participants with CKD, participants without records for blood tests were further excluded. Meanwhile, to reduce the adverse causality between systemic inflammatory indicators and CKD mortality, participants who died within two years were excluded. The specific inclusion and exclusion criteria are summarized in **Figure 1**.

### Definition of systemic inflammatory-related indicators

Blood tests were collected by using the automated hematology analysis devices in each cycle of the survey. Considering the clinical accessibility, generalizability, established validity, and comprehensive reflection of the immune as well as inflammatory status of the CKD population, four systemic inflammatory-related indicators of interest were analyzed, including the SII, NLR, PLR, and LMR. Previous studies have validated their effectiveness in predicting outcomes and informing treatment strategies, making them attractive candidates for investigation in our specific research context.


$$\text{SII}=\frac{\text{Platelet}*\text{Neutrophil}}{\text{Lymphocyte}}$$

$$\text{NLR}=\frac{\text{Neutrophil}}{\text{Lymphocyte}}$$

$$\text{PLR}=\frac{\text{Platelet}}{\text{Lymphocyte}}$$

$$\text{LMR}=\frac{\text{Lymphocyte}}{\text{Monocyte}}$$

### CKD definition

The participants with CKD were diagnosed based on two aspects. On the one hand, the participants had a self-reported history of CKD. On the other hand, eGFR< 60 mL/min/1.73m^2^ was additionally used to diagnose the CKD. The eGFR was calculated according to the Chronic Kidney Disease Epidemiology Collaboration algorithm (27).

### Covariates definition

The selection of study variables was based on previous literature in evaluating the survival of CKD. A series of covariates were controlled including the socioeconomic characteristics included sex (male or female), age at interview, race (Hispanic, non-Hispanic white, non-Hispanic black, and other race), marital status (not married, married or living with partner), educational level (≤ high school, college, and > college), and family income-poverty ratio (<1.3, 1.3 – 3.5, and >3.5). Besides, the personalized habits and comorbidities include smoking status (never, now, and ever), diabetes mellitus, hypertension, hyperlipidemia, congestive heart failure, and self-reported history of dialysis during the past 12 months. In addition, physical and laboratory examinations included body mass index (BMI), estimated glomerular filtration rate (eGFR), blood urea nitrogen (BUN), alanine transaminase (ALT), aspartate transaminase (AST), glycohemoglobin (HbA1c), and albumin (ALB) were controlled.

### Study outcome

The primary outcome was the all-cause mortality of participants with CKD. The survival data for the population were obtained from the NHANES public-use linked mortality file as of December 31, 2019, which was correlated with the National Center for Health Statistics (NCHS) with the National Death Index (NDI) through a probability matching algorithm. Additionally, the ICD-10 (International Statistical Classification of Diseases, 10th revision) was applied to identify the underlying causes of mortality. The primary mortality outcome considered in our study was all-cause mortality. The duration of mortality follow-up was calculated from the date when the diagnosis of CKD was initially taken to either the date of the patient’s death or December 31, 2019 (28).

### Statistical analysis

The continuous variables were presented as mean ± standard deviation (SD). The categorical variables were presented as numbers (percentages, %). The demographic characteristics were compared by using the Student’s t-test or One-way analysis of variance for continuous variables and the Chi-squared test was conducted for categorical variables, respectively. Univariate and multivariable Cox regression analyses were conducted to evaluate the associations between varied inflammatory indicators with all-cause mortality of the CKD population. Model 1 served as the crude model with no adjustments. Additionally, adjustments for age, sex, and race were made in Model 2. In the fully adjusted model, covariates including age, sex, race, marital status, educational level, family income-poverty ratio, smoking status, hypertension, hyperlipidemia, diabetes mellitus, congestive heart failure, BMI, eGFR, BUN, ALT, AST, HbA1c, and ALB were adjusted. Meanwhile, Kaplan-Meier (KM) curves were utilized to display the different survival probabilities among the CKD population with varied levels of systemic inflammatory indicators. To assess the potential nonlinear associations of systemic inflammatory indicators with CKD mortality and to capture the variation in risk across the entire continuum of relations, restricted cubic splines (RCS) with 4 knots were employed. Compared with the conventional simple imputation, the random forest imputation method can not only consider the relationships between variables by using a predictive model, leading to more accurate imputations but also provide estimates of variable importance, aiding in understanding the relative importance of different features in the imputation process (29, 30). Thus, to strike a balance between maximizing data completeness and minimizing potential biases introduced by imputation, variables with missing data were interpolated using the random forest interpolation method.

The area under the curve (AUC) of time-dependent receiver operating characteristic (ROC) and concordance index (C-index) were used to compare the predictive accuracy of each inflammatory indicator in predicting the survival of the CKD population.

We performed the sensitivity analyses to check the robustness of the main findings. As dialysis might significantly influence the levels of systemic inflammatory indicators as well as the survival of the CKD population, we reevaluated the association between systemic inflammatory indicators with the all-cause mortality of the CKD population by further adjusting the covariate of history of renal dialysis during the past 12 months.

All statistical analyses were conducted using the R software (version 4.3.2). A two-tailed P-value of< 0.05 was considered as statistically significant.

## Results

### Baseline information of the participants with CKD

There were 6,880 participants included in the current study during the ten cycles of surveys (1999 – 2018). The mean age of the study population was 67.03 years old. A slightly higher proportion of females than males was observed (3,490 cases vs. 3,390 cases). More than half of the study population was non-Hispanic White (3,571 cases, 51.99%). Less than 20% of the population has an educational level of college. Over 60% of the population had a history of hypertension and nearly half of the study population had hyperlipidemia condition. There were 13.72% and 47.9% of the participants still smoking or drinking in the year of the interview. Compared with the survivors during the follow-up, non-survivors presented characteristics of males, older age, lower BMI, lower levels of eGFR, ALB but higher BUN and HbA1c (all p<0.001). Additionally, survivors showed lower serum levels of SII (541.34 ± 368.46 vs. 666.25 ± 522.15, p<0.001), NLR (2.25 ± 1.32 vs. 2.77 ± 1.62, p<0.001), PLR (127.65 ± 54.50 vs. 144.13 ± 70.41, p<0.001), but higher LMR (3.99 ± 1.99 vs. 3.38 ± 2.08, p<0.001). The study population with clinical characteristics of older age and non-Hispanic whites tended to present higher levels of pro-inflammation conditions when compared with other groups. The specific clinical information of the study population can be found in **Table 1** and the comparisons of varied levels of quartile of inflammatory indicators among the CKD population can be found in **Supplementary Tables S1–S4**.

**Table 1:** The baseline information of the CKD population in this study.

Variable	Total (n = 6,880)	Survivors (n = 4,291)	Non-survivors (n = 2,589)	P ^a^
Age	67.03 ± 13.98	62.29 ± 14.23	74.87 ± 9.22	**<.001**
BMI	29.65 ± 6.59	30.15 ± 6.75	28.81 ± 6.24	**<.001**
eGFR	50.62 ± 25.79	54.44 ± 28.33	44.29 ± 19.33	**<.001**
BUN	7.02 ± 3.28	6.49 ± 2.90	7.90 ± 3.68	**<.001**
ALT	22.28 ± 27.56	22.95 ± 17.28	21.17 ± 39.02	**0.009**
AST	25.17 ± 12.83	25.14 ± 13.22	25.23 ± 12.15	0.776
HbA1c	6.05 ± 1.18	6.03 ± 1.17	6.07 ± 1.20	0.171
ALB	41.42 ± 3.38	41.71 ± 3.31	40.95 ± 3.44	**<.001**
SII	588.35 ± 436.92	541.34 ± 368.46	666.25 ± 522.15	**<.001**
NLR	2.45 ± 1.46	2.25 ± 1.32	2.77 ± 1.62	**<.001**
PLR	133.85 ± 61.49	127.65 ± 54.50	144.13 ± 70.41	**<.001**
LMR	3.76 ± 2.05	3.99 ± 1.99	3.38 ± 2.08	**<.001**
Sex				**<.001**
Female	3,490 (50.73)	2,263 (52.74)	1,227 (47.39)	
Male	3,390 (49.27)	2,028 (47.26)	1,362 (52.61)	
Race				**<.001**
Hispanics	1,040 (15.12)	746 (17.39)	294 (11.36)	
Non-Hispanics White	3,571 (51.9)	1,907 (44.44)	1,664 (64.27)	
Non-Hispanics Black	1,920 (27.91)	1,357 (31.62)	563 (21.75)	
Other	349 (5.07)	281 (6.55)	68 (2.63)	
Education level				**<.001**
≤ High school	3,862 (56.13)	2,162 (50.38)	1,700 (65.66)	
College	1,766 (25.67)	1,225 (28.55)	541 (20.90)	
> College	1,252 (18.2)	904 (21.07)	348 (13.44)	
Marital status				**<.001**
Not married	3,202 (46.54)	1,839 (42.86)	1,363 (52.65)	
Married or living with partner	3,678 (53.46)	2,452 (57.14)	1,226 (47.35)	
Family income-poverty ratio				**<.001**
<1.3	1,924 (27.97)	1,139 (26.54)	785 (30.32)	
1.3-3.5	3,291 (47.83)	1,945 (45.33)	1,346 (51.99)	
>3.5	1,665 (24.2)	1207 (28.13)	458 (17.69)	
Hypertension				**<.001**
No	2,398 (34.85)	1,600 (37.29)	798 (30.82)	
Yes	4,482 (65.15)	2,691 (62.71)	1,791 (69.18)	
Hyperlipidemia				**0.023**
No	3,554 (51.66)	2,171 (50.59)	1,383 (53.42)	
Yes	3,326 (48.34)	2,120 (49.41)	1,206 (46.58)	
Diabetes mellitus				**<.001**
No	4,957 (72.05)	3,168 (73.83)	1,789 (69.10)	
Yes	1,736 (25.23)	1,004 (23.40)	732 (28.27)	
Borderline	187 (2.72)	119 (2.77)	68 (2.63)	
CHF				**<.001**
No	6,187 (89.93)	4,013 (93.52)	2,174 (83.97)	
Yes	693 (10.07)	278 (6.48)	415 (16.03)	
Smoking status				**<.001**
Never	3,418 (49.68)	2,283 (53.20)	1,135 (43.84)	
Now	944 (13.72)	641 (14.94)	303 (11.70)	
Ever	2,518 (36.6)	1,367 (31.86)	1,151 (44.46)	
Alcohol Use				**<.001**
Never	1,892 (27.64)	944 (22.18)	948 (36.62)	
Now	3,279 (47.9)	2,304 (54.14)	975 (37.66)	
Ever	1,674 (24.46)	1,008 (23.68)	666 (25.72)	

Continuous variables are reported as the mean value with the standard deviation (SD) and categorical variables are reported as the frequency with the percentage in parentheses. ^a^ Bold value means statistically significant.

CKD, chronic kidney disease; BMI, body mass index; eGFR, estimated glomerular filtration rate; BUN, blood urea nitrogen; ALT, alanine transaminase; AST, aspartate transaminase; HbA1c, glycosylated hemoglobin; ALB, albumin; SII, systemic immune-inflammation index; NLR, neutrophil-to-lymphocyte ratio; PLR, platelet-to-lymphocyte ratio; LMR, lymphocyte-to-monocyte ratio. CHF, congestive heart failure.

### Association between systemic inflammatory indicators with all-cause mortality of CKD

**Figure 2:**
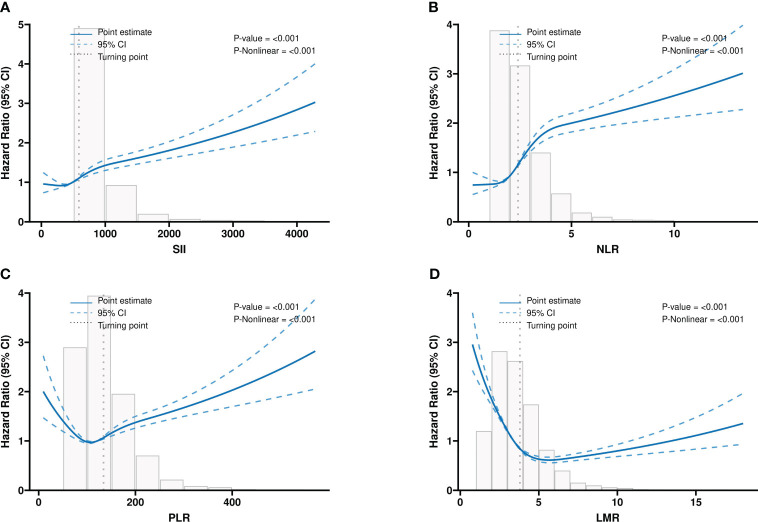
The multivariable-adjusted restricted cubic spline (RCS) curves show dose-effect relationships between different systemic inflammatory indicators with the survival of the CKD population. **(A)** SII; **(B)** NLR; **(C)** PLR; **(D)** LMR. CKD: chronic kidney disease; SII: systemic immune-inflammation index; NLR: neutrophil-to-lymphocyte ratio; PLR: platelet-to-lymphocyte ratio; LMR: lymphocyte-to-monocyte ratio. Adjusted for the effects of age, Sex, race, marital status, educational level, family income-poverty ratio, smoking status, hypertension, hyperlipidemia, diabetes mellitus, CHF, BMI, eGFR, BUN, ALT, AST, HbA1c, and ALB.

The RCS analysis suggested non-linear associations of four systemic inflammatory indicators with the all-cause mortality of participants with CKD (All p< 0.001). The inflection point of the RCS curve was identified at 588 in SII, 134 in PLR, 2.4 in NLR, and 3.8 in LMR, respectively (**Figure 2**).

**Figure 3:**
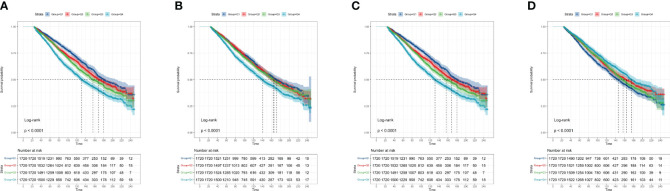
The Kaplan-Meier curves display the association between different systemic inflammatory indicators with the survival of the CKD population. **(A)** SII; **(B)** NLR; **(C)** PLR; **(D)** LMR. CKD: chronic kidney disease; SII: systemic immune-inflammation index; NLR: neutrophil-to-lymphocyte ratio; PLR: platelet-to-lymphocyte ratio; LMR: lymphocyte-to-monocyte ratio.

Besides, the four systemic inflammatory indicators were calculated as categorical variables. The KM curves showed significantly different survival patterns among participants with varied quartiles of systemic inflammatory indicators (All p< 0.0001) (**Figure 3**). Consistently, the higher the quartile of systemic inflammatory indicators (SII, PLR, and NLR) the participants were, the lower survival probabilities were observed. By contrast, the high quartile of levels of LMR predicted an increased survival rate in the CKD population.

**Figure 4:**
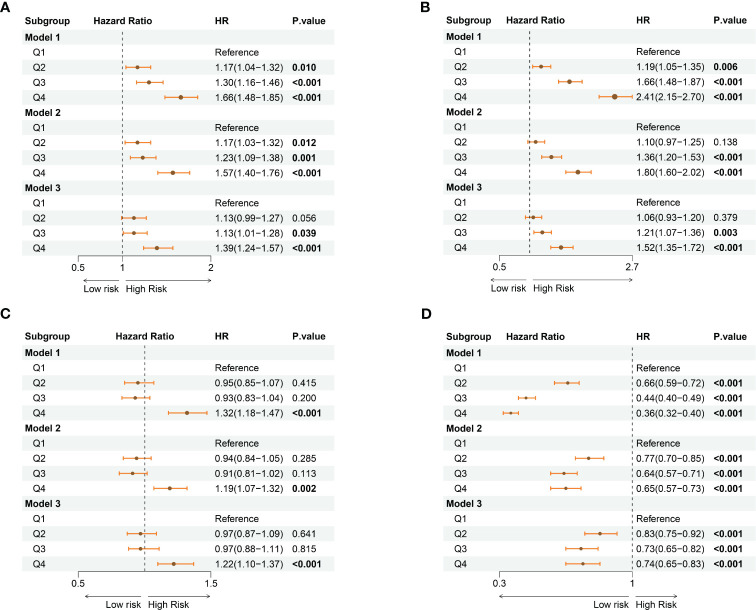
The forest plots show the association between varied levels of quartiles of systemic inflammatory indicators with the risk for all-cause mortality among the CKD population. **(A)** SII; **(B)** NLR; **(C)** PLR; **(D)** LMR. CKD: chronic kidney disease; SII: systemic immune-inflammation index; NLR: neutrophil-to-lymphocyte ratio; PLR: platelet-to-lymphocyte ratio; LMR: lymphocyte-to-monocyte ratio. Model 1: no adjustments; Model 2: adjusted for age, sex, and race Model 3: adjusted for age, sex, race, marital status, educational level, family income-poverty ratio, smoking status, hypertension, hyperlipidemia, diabetes mellitus, congestive heart failure, BMI, eGFR, BUN, ALT, AST, HbA1c, and ALB.

Furthermore, we conducted multivariate Cox regression analyses to evaluate the independent role of varied systemic inflammatory indicators in all-cause mortality among CKD participants (**Supplementary Tables S5–S8**). As shown in **Figure 4**, SII was observed as a significant predictor in all-cause mortality of CKD participants [hazard ratio (HR) = 1.13, 95% confidence interval (CI): 0.99-1.27 in 2^nd^ quartile, p = 0.056; HR = 1.13, 95%CI: 1.00-1.28 in 3^rd^ quartile, p = 0.039; HR =1.39, 95%CI: 1.24-1.57 in 4^th^ quartile, p< 0.001] (**Figure 4A**). Similarly, high quartile levels of NLR were observed as a significant predictor in all-cause mortality of CKD participants [HR = 1.21, 95%CI: 1.07-1.36 in 3^rd^ quartile, p = 0.003; HR = 1.52, 95%CI: 1.35 – 1.72 in 4^th^ quartile, p< 0.001] (**Figure 4B**). Regarding the PLR, participants at the fourth quartile of the index showed a significantly higher risk for mortality (HR = 1.22, 95%CI: 1.10 – 1.37, p< 0.001) (**Figure 4C**). Additionally, higher quartile levels of LMR showed a protectable role in the survival of CKD participants HR = 0.83, 95%CI: 0.75-0.92 in 2^nd^ quartile, p< 0.001; HR = 0.73, 95%CI: 0.65-0.82 in 3^rd^ quartile, p< 0.001; HR = 0.74, 95%CI: 0.65-0.83 in 4^th^ quartile, p< 0.001] (**Figure 4D**).

### Predictive accuracy of systemic inflammatory indicators with survival of CKD population

**Figure 5:**
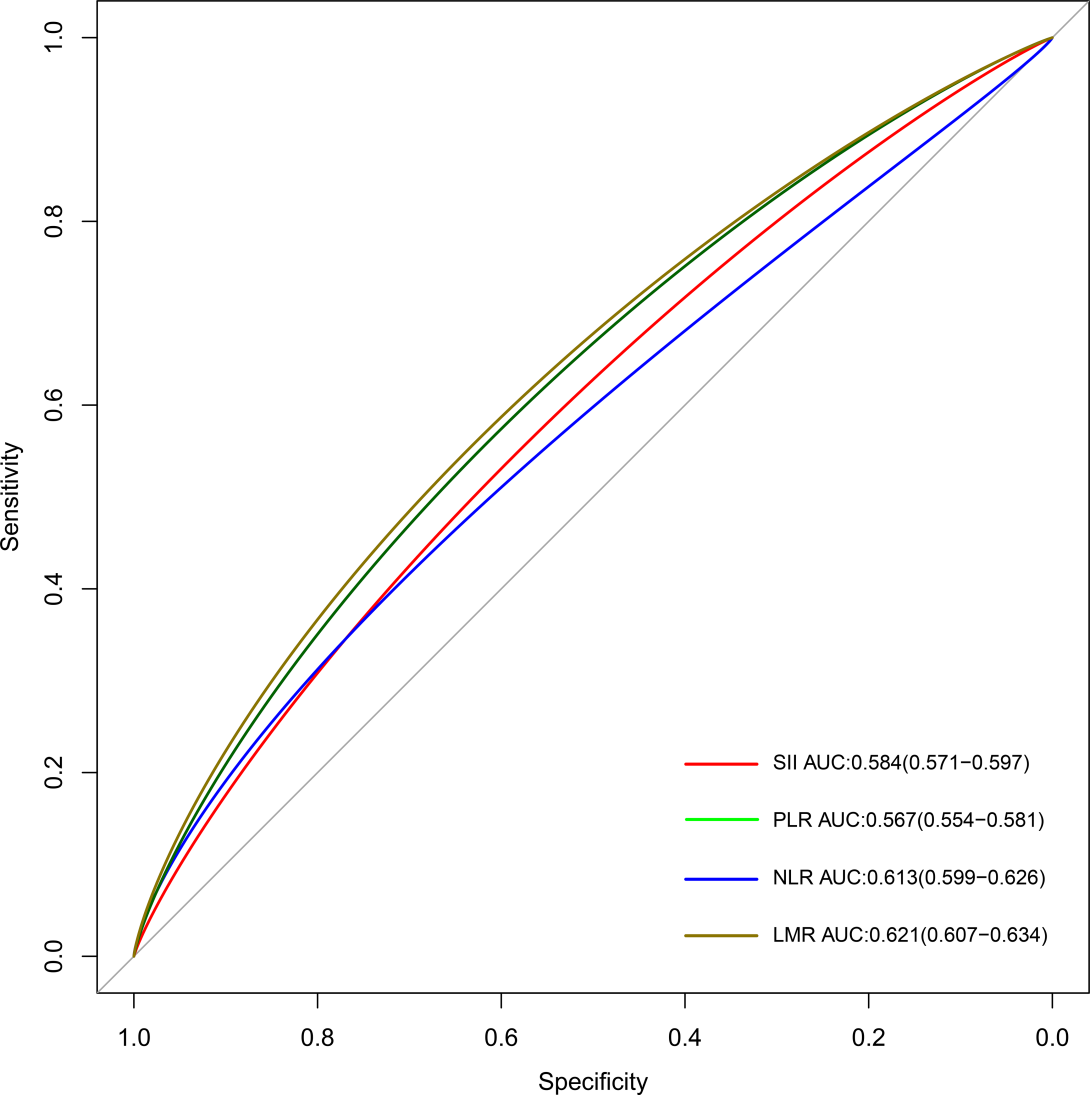
The time-dependent ROC curves for displaying the predictive abilities of different systemic inflammatory indicators. SII: systemic immune-inflammation index; NLR: neutrophil-to-lymphocyte ratio; PLR: platelet-to-lymphocyte ratio; LMR: lymphocyte-to-monocyte ratio; ROC: receiver operating characteristic.

The AUCs of time-dependent ROCs showed that the LMR index maintained the highest predictive value in determining the all-cause mortality of the CKD population (AUC=0.621), followed by the NLR index (AUC=0.613), compared with the rest indicators (**Figure 5** and **Supplementary Table S9**). Consistently, the highest C-index was observed in the LMR index (C-index = 0.624) but the lowest C-index was observed in the PLR index (C-index = 0.537) for predicting the survival of the CKD population (**Supplementary Table S5**). With the combination of other significant clinical factors, the C-index reached 0.789 in the LMR index, 0.785 in the NLR index, 0.779 in SII, and 0.768 in PLR, respectively (**Supplementary Table S5**).

### Stratified and sensitive analyses

There were some interactions among the levels of ALB, family income poverty ratio in SII, NLR, and LMR, and ALT in the PLR index (**Supplementary Figures S1–S4**). The subgroup analyses showed that features of sex, age at the interview, comorbidity status, family income-poverty ratio, and eGFR status would affect the predictive value of systemic inflammatory indicators on the survival of the CKD population (**Supplementary Figures S1–S4**). In addition, the LMR index showed a more pronounced predictive value in the stage III CKD subpopulation, while SII showed a stronger association with the stage IV CKD subpopulation (**Supplementary Tables S10–S11**). However, the predictive values of systemic inflammatory indicators were compromised in the stage V CKD subpopulation (**Supplementary Table S12**). To verify the robustness of the main findings, we further controlled the clinical information on renal dialysis during the past 12 months before the survey. Consistently, the sensitive analysis supported the significant association between systemic inflammatory indicators and with survival of the CKD population (**Supplementary Tables S13–S16**).

## Discussion

In the current study, we determined positive associations between systemic inflammatory indicators with the all-cause mortality of the CKD population. In particular, NLR and LMR showed the leading prediction value in clinical outcomes of the CKD population. To the best of our knowledge, this is one of few studies to evaluate the role of systemic inflammatory indicators in the prognosis of the CKD population based on one large-scale, population-based cohort.

To date, CKD remains one of the leading diseases for causing additional comorbidities and premature mortality. Determining new simple prognostic biomarkers would help clinicians make tailored management decisions on this population. Chronic inflammation disorders have been observed in the CKD population, especially in the late-stage group. Therefore, systemic inflammatory indicators derived from frequent blood tests were considered to be a feasible tool in predicting the survival of the CKD population. For instance, one single center-based study revealed that systemic inflammatory indicators presented optimal survival prediction value in acute coronary syndrome patients with CKD, with AUCs between 0.638 and 0.706. Notably, in another large multi−center longitudinal study settled in China, Lai et al. further validated the utility of SII in predicting the total and cause−specific mortality among patients with CKD. In our works, we also observed strong correlations between high levels of pro-inflammatory condition indicators with worse survival probabilities among the CKD population.

Interestingly, NLR and LMR but not SII showed the best predictive powers in predicting the all-cause mortality of the CKD population. The NLR, a surrogate marker for systemic inflammation, has recently gained increasing public interest. Emerging evidence suggested that NLR was associated with several comorbidities, including insulin resistance and CVD (31, 32). Consistently, high levels of NLR were also determined to be related to the occurrence, and poor nutritional status as well as worse prognosis of CKD (33–37). Of note, Yoshitomi and colleagues observed that CKD patients with high NLR showed a nearly 1.7-fold increased risk for poor renal outcome when compared with the low NLR group in Japan (35). Similar associations were also determined in end-stage CKD patients (37). Nevertheless, the single-center-based experience with a small sample size limited the generalization of findings on this topic. In the current study, we filled this gap and further validated the utility of monitoring the serum NLR in predicting the prognosis of the U.S. population with CKD. Historically, the main possible underlying mechanism regarding the relationship between NLR and the prognosis of the CKD population was thought to be an increase in chronic inflammation. Previous studies have demonstrated a strong association between NLR and inflammatory markers such as TNF-α, CRP, and albumin, suggesting that high NLR reflects chronic inflammatory conditions in CKD patients (38, 39). Novelty, we observed that the high levels of LMR showed a protectable role in the clinical outcome of the CKD population. While the studies evaluating the role of LMR in the prognosis of CKD were limited, compelling evidence has proved the consistent beneficial role of high levels of LMR in other diseases, including but not limited to stroke and cancers (40–42). Recently, peripheral lymphocyte counts have been discovered to have a cell protective effect and contribute to cellular function improvement (42, 43). However, the peripheral monocytes and neutrophils could induce increasing levels of matrix metalloproteinase-9 (MMP-9) and further cause systemic inflammation (44). Furthermore, it was reported that neutrophils could induce free oxygen radicals (45, 46), which was speculated to be associated with reduced renal function and subsequent worse clinical outcomes. different from the promising predictive roles of LMR and NLR on the progress of CKD, only the last quartile level of PLR was observed to be associated with a worse prognosis of the CKD population. Elevated serum levels of platelet indicated endothelial injury and chronic inflammatory condition (47). However, platelet abnormalities were frequently observed in the CKD population, which showed complex implications in the pathophysiology progress of CKD (47). The altered platelet function would result in either platelet hyper- or hypo-reactivity, which might contribute to thrombotic or hemorrhagic complications in CKD (47–49). A recent meta-analysis showed antiplatelet therapy would reduce myocardial infarction and increase major bleeding. However, it did not appear to reduce causes and cardiovascular death among people with CKD and those treated with dialysis (50). A deep understanding of the etiology underlying platelet dysfunction during the CKD progression may contribute to the design of targeted novel antiplatelet treatment strategies, specifically tailored to patients with CKD (47, 48, 50, 51).

There are some strengths worth highlighting. First, this is a prospective, representative, large-scale population-based study based on the U.S. population. Second, we systemic analyzed the predictive value of four systemic inflammatory indicators for all-cause mortality among CKD participants, which provides new evidence for the pivotal role of systemic inflammatory disorders among CKD participants. Third, we excluded the participants who died within 2 years after the interview to reduce the causality bias. Besides, the sensitive analysis also supported the main findings we determined.

Admittedly, some limitations need to be addressed in future works. First, while a series of covariates have been adjusted to determine the association between systemic inflammatory indicators with the survival of CKD participants, some residual confounders such as the duration and cycles of the dialysis, and the history of kidney transplantation with the immunosuppression therapy. As the majority of the CKD population in our study was at stage III, the determined associations between systemic inflammatory indicators and survival of the CKD population might not be impaired. Nevertheless, our findings should be interpreted cautiously due to the diagnosis of CKD was based on the self-reported condition and baseline levels of eGFR. Second, the data was derived from the NHANES program, which could not fully reflect the prevalence and stage of the CKD population. The predictive value of systemic inflammatory indicators in the CKD population at different stages is worth investigating in future works. Moreover, we only analyzed the data of systemic inflammatory indicators at the baseline of the surveys, whether the trajectories of these indicators showed a more pivotal role in predicting the survival of CKD participants needs further exploration. Future well-designed, longitudinal, prospective studies are warranted to validate our findings.

## Conclusion

In this study, we observed the significant associations between systemic inflammatory indicators with all-cause mortality of CKD in the U.S. population. Besides, the SII showed the highest prediction value in identifying the high-risk subpopulation with CKD when compared with rest indices. Our findings would help the nephrologists to make dynamic monitoring of the long-term follow-up among the CKD population with simple serum inflammatory levels.

## Data availability statement

The original contributions presented in the study are included in the article/**Supplementary Material**. Further inquiries can be directed to the corresponding authors.

## Ethics statement

All survey protocols were approved by the National Center for Health Statistics Review Board in the U.S.A. All participants provided written informed consent before participation.

## Author contributions

YC: Conceptualization, Data curation, Formal analysis, Funding acquisition, Investigation, Methodology, Project administration, Resources, Software, Supervision, Validation, Visualization, Writing – original draft, Writing – review & editing. YN: Conceptualization, Data curation, Formal analysis, Investigation, Methodology, Project administration, Resources, Validation, Visualization, Writing – original draft, Writing – review & editing. JW: Conceptualization, Data curation, Formal analysis, Investigation, Methodology, Visualization, Writing – original draft, Writing – review & editing. CL: Conceptualization, Formal analysis, Investigation, Project administration, Resources, Software, Visualization, Writing – original draft, Writing – review & editing. LZ: Conceptualization, Formal analysis, Investigation, Project administration, Resources, Software, Validation, Visualization, Writing – original draft, Writing – review & editing. BZ: Conceptualization, Investigation, Project administration, Resources, Software, Visualization, Writing – original draft, Writing – review & editing. YM: Data curation, Methodology, Conceptualization, Resources, Visualization, Writing – original draft, Writing – review & editing. TL: Conceptualization, Data curation, Formal analysis, Funding acquisition, Investigation, Methodology, Project administration, Resources, Software, Supervision, Validation, Visualization, Writing – original draft, Writing – review & editing. XL: Conceptualization, Data curation, Formal analysis, Funding acquisition, Investigation, Methodology, Project administration, Resources, Software, Supervision, Validation, Visualization, Writing – original draft, Writing – review & editing.
