# Supplementary material for: Association between systemic inflammatory indicators with the survival of chronic kidney disease: a prospective study based on NHANES
Source: Front Immunol. 2024 Apr 8;15:1365591. doi: 10.3389/fimmu.2024.1365591 (PMC11033417; doi:10.3389/fimmu.2024.1365591)
Supplement: Supplementary file 1 [file DataSheet_1.docx]

**Supplementary files**

**Association between systemic inflammatory indicators with the survival of chronic kidney disease: A prospective study based on NHANES**

Yuan Chen ^a^, Yanfang Nie ^a^, Jiaying Wu ^a^, Chunsheng Li ^a^, Lu Zheng ^a^, Bixiu Zhu ^a^, Hanlei Song ^b*^, Tao Ling ^c*^, Xiaozhu Liu ^d*^

^a^ Department of Nephrology, Taizhou Central Hospital (Taizhou University

Hospital), Zhejiang, China

^b^ Department of Nephrology, Ruian People’s Hospital, Ruian, China

^c^ Department of Pharmacy, Suqian First Hospital, Suqian, China

^d^ Department of Critical Care Medicine, Beijing Shijitan Hospital, Capital Medical University, Beijing, China.

*Corresponding Authors:

Prof. Hanlei Song is to be contacted at the Department of Pharmacy, Suqian First Hospital, Suqian, China. E-mail address: [shlyou@126.com](mailto:shlyou@126.com).

Prof. Tao Ling is to be contacted at the Department of Pharmacy, Suqian First Hospital, Suqian, China. E-mail address: [ling_tao2022@163.com](mailto:ling_tao2022@163.com).

Prof. Xiaozhu Liu is to be contacted at the Department of Critical Care Medicine, Beijing Shijitan Hospital, Capital Medical University, Beijing, China. E-mail address: xiaozhuliu2021@163.com.

**Figure S1.** The forest plot shows the subgroup analysis with interaction between variables of SII.


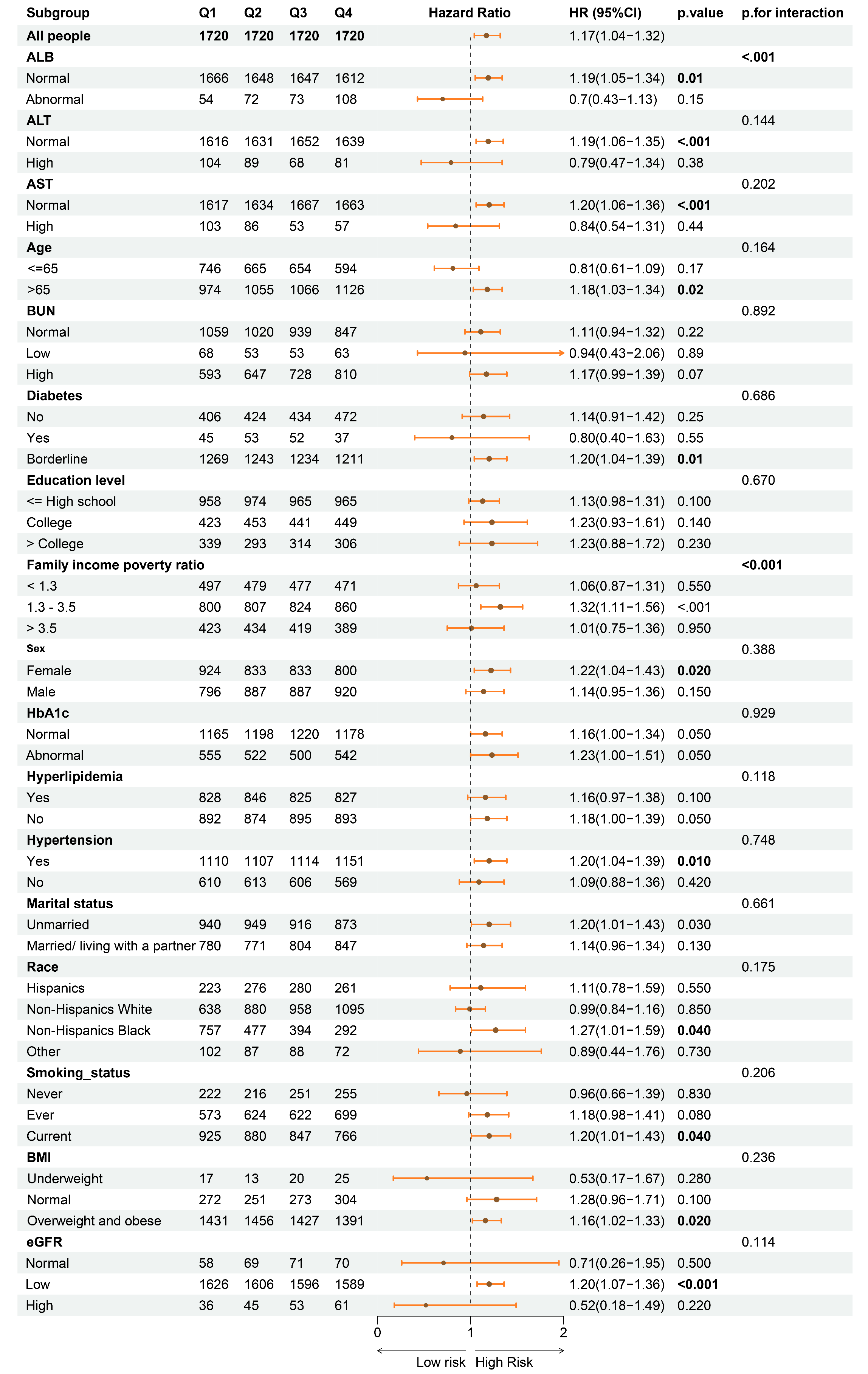


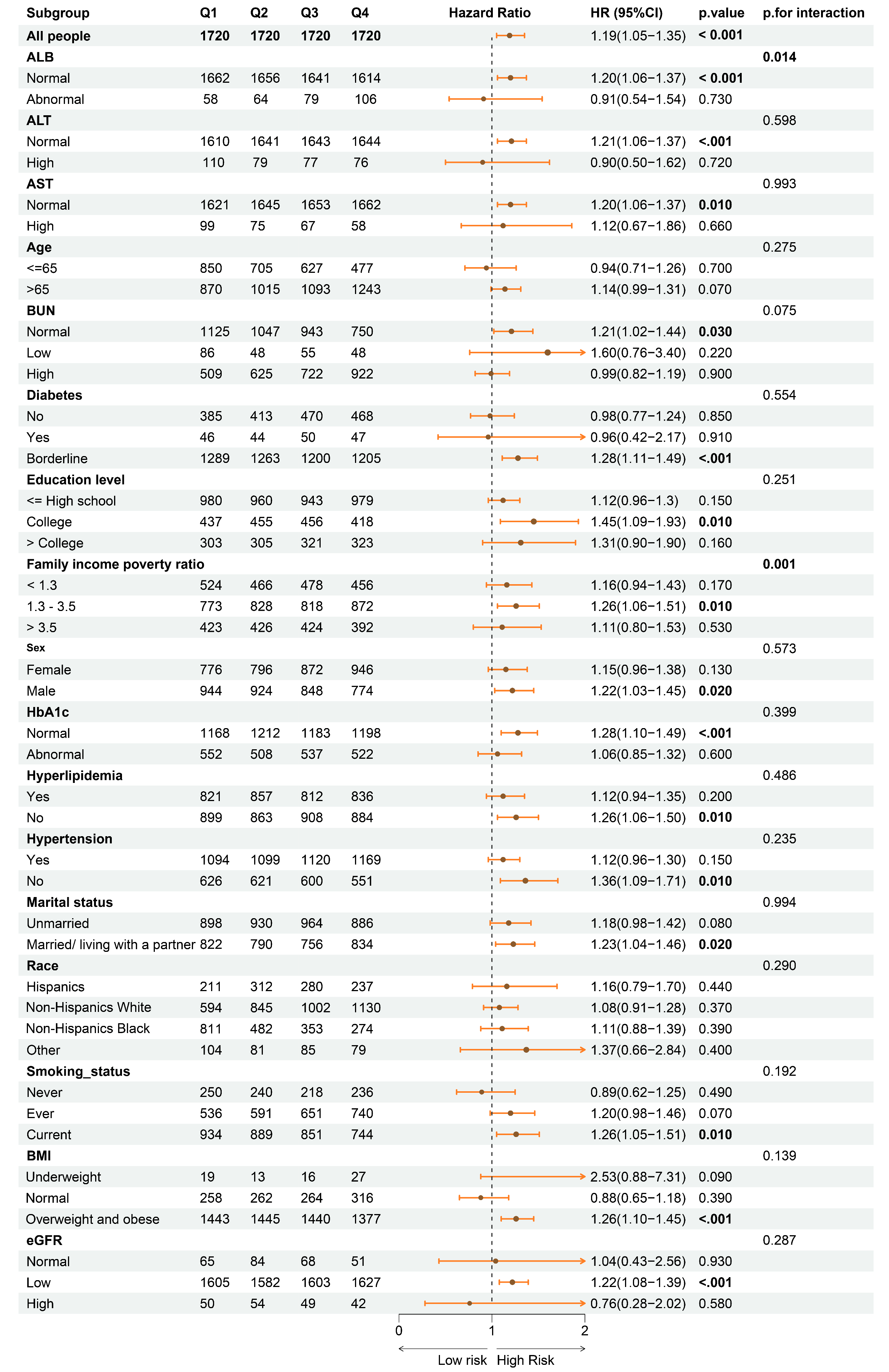
**Figure S2.** The forest plot shows the subgroup analysis with interaction between variables of NLR.


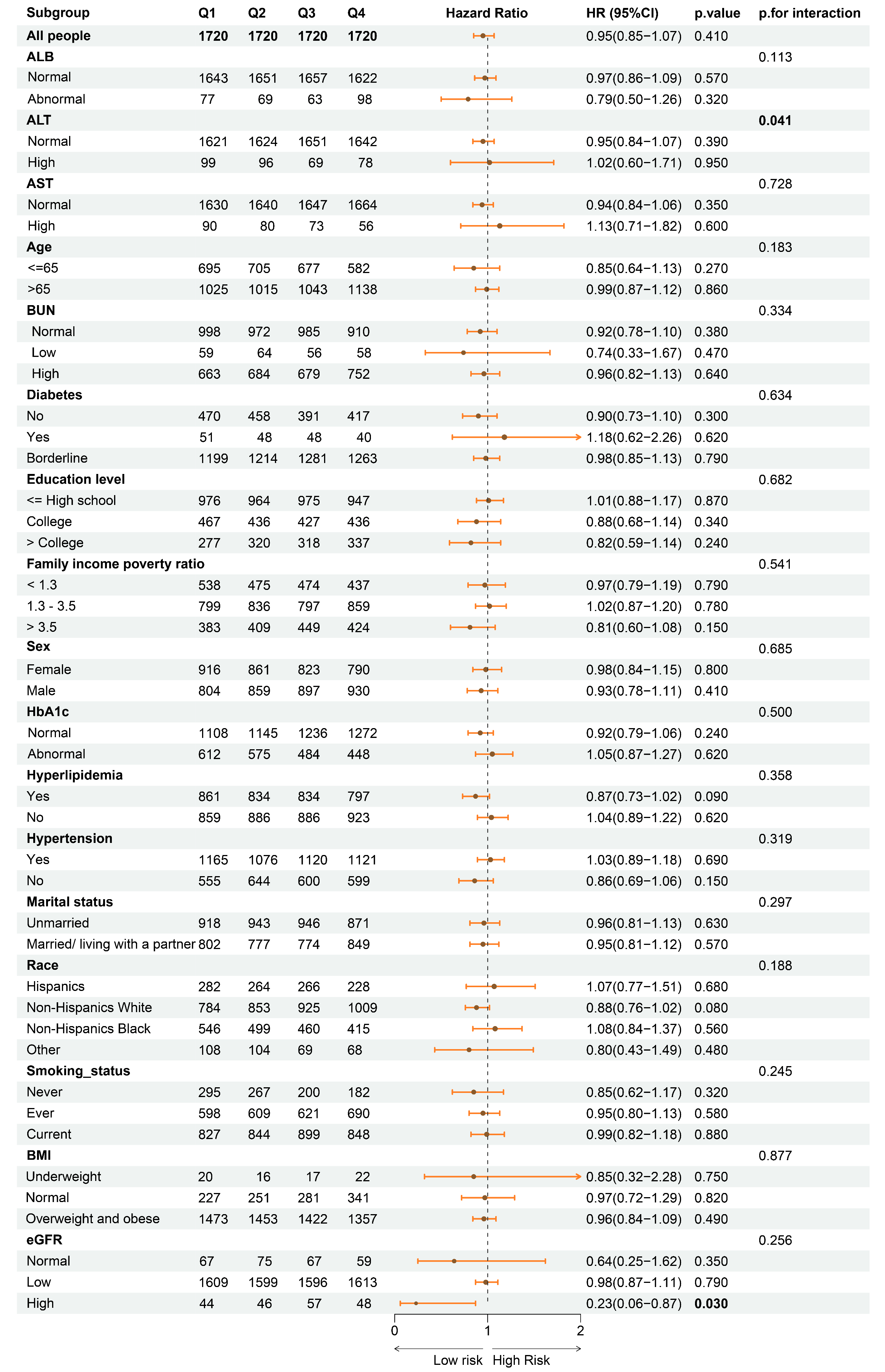
**Figure S3.** The forest plot shows the subgroup analysis with interaction between variables of PLR.

**Figure S4.** The forest plot shows the subgroup analysis with interaction between variables of LMR.


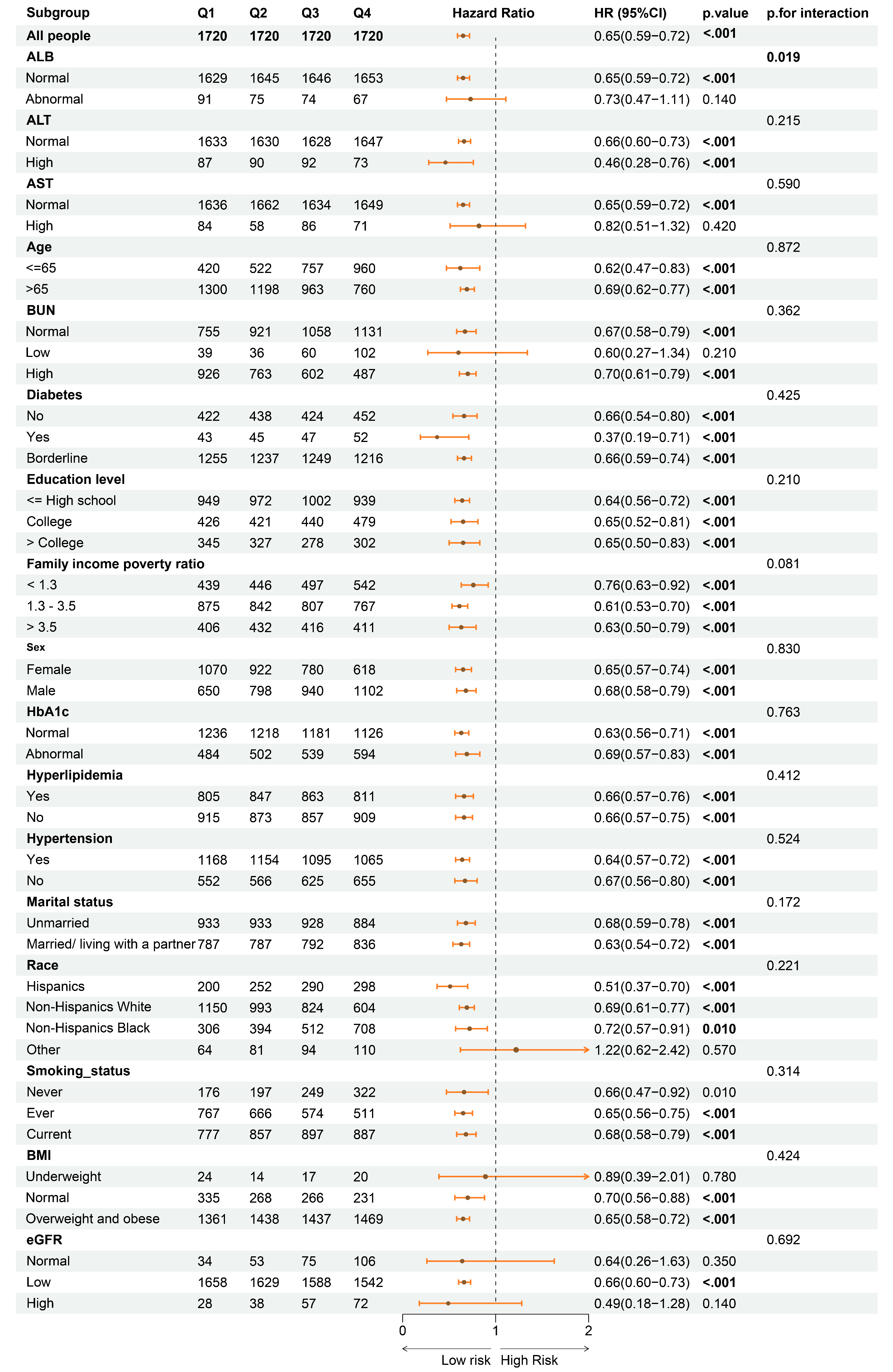


**Table S1**. The comparisons between each quartile level of SII.

| **Variable** | **Total (n = 6880)** | **SII** | | | | **P** ^a^ |
| --- | --- | --- | --- | --- | --- | --- |
|  |  | **Q1 (n = 1720)** | **Q2 (n = 1720)** | **Q3 (n = 1720)** | **Q4 (n = 1720)** |  |
| **Age** | 67.03 ± 13.98 | 66.12 ± 13.46 | 66.45 ± 14.17 | 67.19 ± 14.23 | 68.34 ± 13.95 | **<.001** |
| **BMI** | 29.65 ± 6.59 | 29.65 ± 6.18 | 29.75 ± 6.34 | 29.67 ± 6.83 | 29.51 ± 6.99 | 0.754 |
| **eGFR** | 50.62 ± 25.79 | 50.29 ± 21.62 | 51.81 ± 25.79 | 50.15 ± 25.08 | 50.23 ± 29.99 | 0.181 |
| **BUN** | 7.02 ± 3.28 | 6.57 ± 2.90 | 6.84 ± 3.02 | 7.18 ± 3.41 | 7.47 ± 3.68 | **<.001** |
| **ALT** | 22.28 ± 27.56 | 22.84 ± 12.81 | 22.51 ± 12.83 | 21.54 ± 21.74 | 22.24 ± 47.31 | 0.557 |
| **AST** | 25.17 ± 12.83 | 26.61 ± 15.89 | 25.54 ± 11.93 | 24.17 ± 11.82 | 24.38 ± 10.97 | **<.001** |
| **HbA1c** | 6.05 ± 1.18 | 6.06 ± 1.18 | 6.04 ± 1.17 | 6.03 ± 1.17 | 6.05 ± 1.20 | 0.893 |
| **ALB** | 41.42 ± 3.38 | 41.63 ± 3.20 | 41.61 ± 3.33 | 41.44 ± 3.34 | 41.01 ± 3.60 | **<.001** |
| **Sex** |  |  |  |  |  | **<.001** |
| **Female** | 3490 (50.73) | 796 (46.28) | 887 (51.57) | 887 (51.57) | 920 (53.49) |  |
| **Male** | 3390 (49.27) | 924 (53.72) | 833 (48.43) | 833 (48.43) | 800 (46.51) |  |
| **Race** |  |  |  |  |  | **<.001** |
| **Hispanics** | 1040 (15.12) | 223 (12.97) | 276 (16.05) | 280 (16.28) | 261 (15.17) |  |
| **Non-Hispanics White** | 3571 (51.9) | 638 (37.09) | 880 (51.16) | 958 (55.70) | 1095 (63.66) |  |
| **Non-Hispanics Black** | 1920 (27.91) | 757 (44.01) | 477 (27.73) | 394 (22.91) | 292 (16.98) |  |
| **Other** | 349 (5.07) | 102 (5.93) | 87 (5.06) | 88 (5.12) | 72 (4.19) |  |
| **Education level** |  |  |  |  |  | 0.552 |
| **≤ High school** | 3862 (56.13) | 958 (55.70) | 974 (56.63) | 965 (56.10) | 965 (56.10) |  |
| **College** | 1766 (25.67) | 423 (24.59) | 453 (26.34) | 441 (25.64) | 449 (26.10) |  |
| **> College** | 1252 (18.2) | 339 (19.71) | 293 (17.03) | 314 (18.26) | 306 (17.79) |  |
| **Marital status** |  |  |  |  |  | **0.044** |
| **Not married** | 3202 (46.54) | 780 (45.35) | 771 (44.83) | 804 (46.74) | 847 (49.24) |  |
| **Married or living with partner** | 3678 (53.46) | 940 (54.65) | 949 (55.17) | 916 (53.26) | 873 (50.76) |  |
| **Family income-poverty ratio** |  |  |  |  |  | 0.415 |
| **<1.3** | 1924 (27.97) | 497 (28.90) | 479 (27.85) | 477 (27.73) | 471 (27.38) |  |
| **1.3-3.5** | 3291 (47.83) | 800 (46.51) | 807 (46.92) | 824 (47.91) | 860 (50.00) |  |
| **>3.5** | 1665 (24.2) | 423 (24.59) | 434 (25.23) | 419 (24.36) | 389 (22.62) |  |
| **Hypertension** |  |  |  |  |  | 0.356 |
| **No** | 2398 (34.85) | 610 (35.47) | 613 (35.64) | 606 (35.23) | 569 (33.08) |  |
| **Yes** | 4482 (65.15) | 1110 (64.53) | 1107 (64.36) | 1114 (64.77) | 1151 (66.92) |  |
| **Hyperlipidemia** |  |  |  |  |  | 0.882 |
| **No** | 3554 (51.66) | 892 (51.86) | 874 (50.81) | 895 (52.03) | 893 (51.92) |  |
| **Yes** | 3326 (48.34) | 828 (48.14) | 846 (49.19) | 825 (47.97) | 827 (48.08) |  |
| **Diabetes mellitus** |  |  |  |  |  | 0.113 |
| **No** | 4957 (72.05) | 1269 (73.78) | 1243 (72.27) | 1234 (71.74) | 1211 (70.41) |  |
| **Yes** | 1736 (25.23) | 406 (23.60) | 424 (24.65) | 434 (25.23) | 472 (27.44) |  |
| **Borderline** | 187 (2.72) | 45 (2.62) | 53 (3.08) | 52 (3.02) | 37 (2.15) |  |
| **CHF** |  |  |  |  |  | **<.001** |
| **No** | 6187 (89.93) | 1559 (90.64) | 1588 (92.33) | 1538 (89.42) | 1502 (87.33) |  |
| **Yes** | 693 (10.07) | 161 (9.36) | 132 (7.67) | 182 (10.58) | 218 (12.67) |  |
| **Smoking status** |  |  |  |  |  | **<.001** |
| **Never** | 3418 (49.68) | 925 (53.78) | 880 (51.16) | 847 (49.24) | 766 (44.53) |  |
| **Now** | 944 (13.72) | 222 (12.91) | 216 (12.56) | 251 (14.59) | 255 (14.83) |  |
| **Ever** | 2518 (36.6) | 573 (33.31) | 624 (36.28) | 622 (36.16) | 699 (40.64) |  |
| **Alcohol Use** |  |  |  |  |  | 0.236 |
| **Never** | 1892 (27.64) | 485 (28.38) | 453 (26.44) | 454 (26.52) | 500 (29.22) |  |
| **Now** | 3279 (47.9) | 789 (46.17) | 834 (48.69) | 853 (49.82) | 803 (46.93) |  |
| **Ever** | 1674 (24.46) | 435 (25.45) | 426 (24.87) | 405 (23.66) | 408 (23.85) |  |

Note: Continuous variables are reported as the mean value with the standard deviation (SD) and categorical variables are reported as the frequency with the percentage in parentheses. ^a^ Bold value means statistically significant

Abbreviation: CKD: chronic kidney disease; SII: systemic immune-inflammation index; BMI: body mass index; eGFR: estimated glomerular filtration rate; BUN: blood urea nitrogen; ALT: alanine transaminase; AST: aspartate transaminase; HbA1c: glycosylated hemoglobin; ALB: albumin; CHF: congestive heart failure.

**Table S2**. The comparisons between each quartile level of NLR.

| **Variable** | **Total (n = 6880)** | **NLR** | | | | **P** ^a^ |
| --- | --- | --- | --- | --- | --- | --- |
|  |  | **Q1 (n = 1720)** | **Q2 (n = 1720)** | **Q3 (n = 1720)** | **Q4 (n = 1720)** |  |
| **Age** | 67.03 ± 13.98 | 64.19 ± 13.91 | 65.81 ± 14.12 | 67.88 ± 13.91 | 70.23 ± 13.24 | **<.001** |
| **BMI** | 29.65 ± 6.59 | 29.87 ± 6.40 | 29.91 ± 6.57 | 29.82 ± 6.85 | 28.98 ± 6.50 | **<.001** |
| **eGFR** | 50.62 ± 25.79 | 52.54 ± 23.43 | 52.34 ± 26.61 | 50.63 ± 25.10 | 46.96 ± 27.49 | **<.001** |
| **BUN** | 7.02 ± 3.28 | 6.25 ± 2.72 | 6.69 ± 2.74 | 7.16 ± 3.42 | 7.97 ± 3.87 | **<.001** |
| **ALT** | 22.28 ± 27.56 | 22.71 ± 12.64 | 22.46 ± 12.39 | 21.98 ± 22.37 | 21.97 ± 47.18 | 0.825 |
| **AST** | 25.17 ± 12.83 | 26.03 ± 13.04 | 25.48 ± 14.09 | 24.91 ± 13.82 | 24.27 ± 9.87 | **<.001** |
| **HbA1c** | 6.05 ± 1.18 | 6.08 ± 1.23 | 6.05 ± 1.17 | 6.03 ± 1.10 | 6.03 ± 1.21 | 0.568 |
| **ALB** | 41.42 ± 3.38 | 41.58 ± 3.25 | 41.76 ± 3.33 | 41.33 ± 3.32 | 41.02 ± 3.58 | **<.001** |
| **Sex** |  |  |  |  |  | **<.001** |
| **Female** | 3490 (50.73) | 944 (54.88) | 924 (53.72) | 848 (49.30) | 774 (45.00) |  |
| **Male** | 3390 (49.27) | 776 (45.12) | 796 (46.28) | 872 (50.70) | 946 (55.00) |  |
| **Race** |  |  |  |  |  | **<.001** |
| **Hispanics** | 1040 (15.12) | 211 (12.27) | 312 (18.14) | 280 (16.28) | 237 (13.78) |  |
| **Non-Hispanics White** | 3571 (51.9) | 594 (34.53) | 845 (49.13) | 1002 (58.26) | 1130 (65.70) |  |
| **Non-Hispanics Black** | 1920 (27.91) | 811 (47.15) | 482 (28.02) | 353 (20.52) | 274 (15.93) |  |
| **Other** | 349 (5.07) | 104 (6.05) | 81 (4.71) | 85 (4.94) | 79 (4.59) |  |
| **Education level** |  |  |  |  |  | 0.650 |
| **≤ High school** | 3862 (56.13) | 980 (56.98) | 960 (55.81) | 943 (54.83) | 979 (56.92) |  |
| **College** | 1766 (25.67) | 437 (25.41) | 455 (26.45) | 456 (26.51) | 418 (24.30) |  |
| **> College** | 1252 (18.2) | 303 (17.62) | 305 (17.73) | 321 (18.66) | 323 (18.78) |  |
| **Marital status** |  |  |  |  |  | **0.035** |
| **Not married** | 3202 (46.54) | 822 (47.79) | 790 (45.93) | 756 (43.95) | 834 (48.49) |  |
| **Married or living with partner** | 3678 (53.46) | 898 (52.21) | 930 (54.07) | 964 (56.05) | 886 (51.51) |  |
| **Family income-poverty ratio** |  |  |  |  |  | **0.035** |
| **<1.3** | 1924 (27.97) | 524 (30.47) | 466 (27.09) | 478 (27.79) | 456 (26.51) |  |
| **1.3-3.5** | 3291 (47.83) | 773 (44.94) | 828 (48.14) | 818 (47.56) | 872 (50.70) |  |
| **>3.5** | 1665 (24.2) | 423 (24.59) | 426 (24.77) | 424 (24.65) | 392 (22.79) |  |
| **Hypertension** |  |  |  |  |  | **0.029** |
| **No** | 2398 (34.85) | 626 (36.40) | 621 (36.10) | 600 (34.88) | 551 (32.03) |  |
| **Yes** | 4482 (65.15) | 1094 (63.60) | 1099 (63.90) | 1120 (65.12) | 1169 (67.97) |  |
| **Hyperlipidemia** |  |  |  |  |  | 0.440 |
| **No** | 3554 (51.66) | 899 (52.27) | 863 (50.17) | 908 (52.79) | 884 (51.40) |  |
| **Yes** | 3326 (48.34) | 821 (47.73) | 857 (49.83) | 812 (47.21) | 836 (48.60) |  |
| **Diabetes mellitus** |  |  |  |  |  | **0.008** |
| **No** | 4957 (72.05) | 1289 (74.94) | 1263 (73.43) | 1200 (69.77) | 1205 (70.06) |  |
| **Yes** | 1736 (25.23) | 385 (22.38) | 413 (24.01) | 470 (27.33) | 468 (27.21) |  |
| **Borderline** | 187 (2.72) | 46 (2.67) | 44 (2.56) | 50 (2.91) | 47 (2.73) |  |
| **CHF** |  |  |  |  |  | **<.001** |
| **No** | 6187 (89.93) | 1596 (92.79) | 1594 (92.67) | 1537 (89.36) | 1460 (84.88) |  |
| **Yes** | 693 (10.07) | 124 (7.21) | 126 (7.33) | 183 (10.64) | 260 (15.12) |  |
| **Smoking status** |  |  |  |  |  | **<.001** |
| **Never** | 3418 (49.68) | 934 (54.30) | 889 (51.69) | 851 (49.48) | 744 (43.26) |  |
| **Now** | 944 (13.72) | 250 (14.53) | 240 (13.95) | 218 (12.67) | 236 (13.72) |  |
| **Ever** | 2518 (36.6) | 536 (31.16) | 591 (34.36) | 651 (37.85) | 740 (43.02) |  |
| **Alcohol Use** |  |  |  |  |  | 0.100 |
| **Never** | 1892 (27.64) | 445 (25.99) | 466 (27.22) | 468 (27.38) | 513 (29.96) |  |
| **Now** | 3279 (47.9) | 814 (47.55) | 827 (48.31) | 826 (48.33) | 812 (47.43) |  |
| **Ever** | 1674 (24.46) | 453 (26.46) | 419 (24.47) | 415 (24.28) | 387 (22.61) |  |

Note: Continuous variables are reported as the mean value with the standard deviation (SD) and categorical variables are reported as the frequency with the percentage in parentheses. ^a^ Bold value means statistically significant

Abbreviation: CKD: chronic kidney disease; NLR: neutrophil-to-lymphocyte ratio; BMI: body mass index; eGFR: estimated glomerular filtration rate; BUN: blood urea nitrogen; ALT: alanine transaminase; AST: aspartate transaminase; HbA1c: glycosylated hemoglobin; ALB: albumin; CHF: congestive heart failure.

**Table S3**. The comparisons between each quartile level of PLR.

| **Variable** | **Total (n = 6880)** | **PLR** | | | | **P** ^a^ |
| --- | --- | --- | --- | --- | --- | --- |
|  |  | **Q1 (n = 1720)** | **Q2 (n = 1720)** | **Q3 (n = 1720)** | **Q4 (n = 1720)** |  |
| **Age** | 67.03 ± 13.98 | 66.35 ± 13.58 | 66.19 ± 14.44 | 66.85 ± 14.07 | 68.71 ± 13.67 | **<.001** |
| **BMI** | 29.65 ± 6.59 | 30.21 ± 6.54 | 29.99 ± 6.70 | 29.77 ± 6.81 | 28.61 ± 6.20 | **<.001** |
| **eGFR** | 50.62 ± 25.79 | 50.78 ± 23.69 | 51.17 ± 26.02 | 51.44 ± 25.80 | 49.09 ± 27.48 | **0.034** |
| **BUN** | 7.02 ± 3.28 | 6.85 ± 3.04 | 6.89 ± 3.05 | 6.99 ± 3.35 | 7.34 ± 3.64 | **<.001** |
| **ALT** | 22.28 ± 27.56 | 22.76 ± 12.45 | 22.38 ± 12.35 | 23.03 ± 51.03 | 20.95 ± 11.24 | 0.119 |
| **AST** | 25.17 ± 12.83 | 25.77 ± 14.51 | 25.43 ± 12.68 | 25.02 ± 13.75 | 24.48 ± 9.87 | 0.022 |
| **HbA1c** | 6.05 ± 1.18 | 6.18 ± 1.27 | 6.09 ± 1.21 | 5.99 ± 1.13 | 5.93 ± 1.07 | **<.001** |
| **ALB** | 41.42 ± 3.38 | 41.51 ± 3.41 | 41.58 ± 3.30 | 41.60 ± 3.28 | 41.01 ± 3.49 | **<.001** |
| **Sex** |  |  |  |  |  | **<.001** |
| **Female** | 3490 (50.73) | 804 (46.74) | 859 (49.94) | 897 (52.15) | 930 (54.07) |  |
| **Male** | 3390 (49.27) | 916 (53.26) | 861 (50.06) | 823 (47.85) | 790 (45.93) |  |
| **Race** |  |  |  |  |  | **<.001** |
| **Hispanics** | 1040 (15.12) | 282 (16.40) | 264 (15.35) | 266 (15.47) | 228 (13.26) |  |
| **Non-Hispanics White** | 3571 (51.9) | 784 (45.58) | 853 (49.59) | 925 (53.78) | 1009 (58.66) |  |
| **Non-Hispanics Black** | 1920 (27.91) | 546 (31.74) | 499 (29.01) | 460 (26.74) | 415 (24.13) |  |
| **Other** | 349 (5.07) | 108 (6.28) | 104 (6.05) | 69 (4.01) | 68 (3.95) |  |
| **Education level** |  |  |  |  |  | 0.181 |
| **≤ High school** | 3862 (56.13) | 976 (56.74) | 964 (56.05) | 975 (56.69) | 947 (55.06) |  |
| **College** | 1766 (25.67) | 467 (27.15) | 436 (25.35) | 427 (24.83) | 436 (25.35) |  |
| **> College** | 1252 (18.2) | 277 (16.10) | 320 (18.60) | 318 (18.49) | 337 (19.59) |  |
| **Marital status** |  |  |  |  |  | **0.038** |
| **Not married** | 3202 (46.54) | 802 (46.63) | 777 (45.17) | 774 (45.00) | 849 (49.36) |  |
| **Married or living with partner** | 3678 (53.46) | 918 (53.37) | 943 (54.83) | 946 (55.00) | 871 (50.64) |  |
| **Family income-poverty ratio** |  |  |  |  |  | **0.003** |
| **<1.3** | 1924 (27.97) | 538 (31.28) | 475 (27.62) | 474 (27.56) | 437 (25.41) |  |
| **1.3-3.5** | 3291 (47.83) | 799 (46.45) | 836 (48.60) | 797 (46.34) | 859 (49.94) |  |
| **>3.5** | 1665 (24.2) | 383 (22.27) | 409 (23.78) | 449 (26.10) | 424 (24.65) |  |
| **Hypertension** |  |  |  |  |  | **0.017** |
| **No** | 2398 (34.85) | 555 (32.27) | 644 (37.44) | 600 (34.88) | 599 (34.83) |  |
| **Yes** | 4482 (65.15) | 1165 (67.73) | 1076 (62.56) | 1120 (65.12) | 1121 (65.17) |  |
| **Hyperlipidemia** |  |  |  |  |  | 0.185 |
| **No** | 3554 (51.66) | 859 (49.94) | 886 (51.51) | 886 (51.51) | 923 (53.66) |  |
| **Yes** | 3326 (48.34) | 861 (50.06) | 834 (48.49) | 834 (48.49) | 797 (46.34) |  |
| **Diabetes mellitus** |  |  |  |  |  | **0.026** |
| **No** | 4957 (72.05) | 1199 (69.71) | 1214 (70.58) | 1281 (74.48) | 1263 (73.43) |  |
| **Yes** | 1736 (25.23) | 470 (27.33) | 458 (26.63) | 391 (22.73) | 417 (24.24) |  |
| **Borderline** | 187 (2.72) | 51 (2.97) | 48 (2.79) | 48 (2.79) | 40 (2.33) |  |
| **CHF** |  |  |  |  |  | **0.005** |
| **No** | 6187 (89.93) | 1534 (89.19) | 1558 (90.58) | 1577 (91.69) | 1518 (88.26) |  |
| **Yes** | 693 (10.07) | 186 (10.81) | 162 (9.42) | 143 (8.31) | 202 (11.74) |  |
| **Smoking status** |  |  |  |  |  | **<.001** |
| **Never** | 3418 (49.68) | 827 (48.08) | 844 (49.07) | 899 (52.27) | 848 (49.30) |  |
| **Now** | 944 (13.72) | 295 (17.15) | 267 (15.52) | 200 (11.63) | 182 (10.58) |  |
| **Ever** | 2518 (36.6) | 598 (34.77) | 609 (35.41) | 621 (36.10) | 690 (40.12) |  |
| **Alcohol Use** |  |  |  |  |  | 0.185 |
| **Never** | 1892 (27.64) | 508 (29.74) | 474 (27.64) | 434 (25.41) | 476 (27.77) |  |
| **Now** | 3279 (47.9) | 791 (46.31) | 818 (47.70) | 856 (50.12) | 814 (47.49) |  |
| **Ever** | 1674 (24.46) | 409 (23.95) | 423 (24.66) | 418 (24.47) | 424 (24.74) |  |

Note: Continuous variables are reported as the mean value with the standard deviation (SD) and categorical variables are reported as the frequency with the percentage in parentheses. ^a^ Bold value means statistically significant

Abbreviation: CKD: chronic kidney disease; PLR: platelet-to-lymphocyte ratio; BMI: body mass index; eGFR: estimated glomerular filtration rate; BUN: blood urea nitrogen; ALT: alanine transaminase; AST: aspartate transaminase; HbA1c: glycosylated hemoglobin; ALB: albumin; CHF: congestive heart failure.

**Table S4**. The comparisons between each quartile level of LMR.

| **Variable** | **Total (n = 6880)** | **LMR** | | | | **P ^a^** |
| --- | --- | --- | --- | --- | --- | --- |
|  |  | **Q1 (n = 1720)** | **Q2 (n = 1720)** | **Q3 (n = 1720)** | **Q4 (n = 1720)** |  |
| **Age** | 67.03 ± 13.98 | 71.62 ± 12.41 | 69.10 ± 13.06 | 65.25 ± 14.30 | 62.14 ± 14.16 | **<.001** |
| **BMI** | 29.65 ± 6.59 | 28.56 ± 6.18 | 29.44 ± 6.32 | 29.89 ± 6.41 | 30.70 ± 7.25 | **<.001** |
| **eGFR** | 50.62 ± 25.79 | 45.73 ± 23.87 | 48.95 ± 24.25 | 52.58 ± 26.98 | 55.21 ± 26.93 | **<.001** |
| **BUN** | 7.02 ± 3.28 | 8.02 ± 3.80 | 7.27 ± 3.23 | 6.67 ± 3.05 | 6.11 ± 2.65 | **<.001** |
| **ALT** | 22.28 ± 27.56 | 22.68 ± 47.39 | 22.21 ± 22.41 | 22.57 ± 12.87 | 21.66 ± 11.23 | 0.703 |
| **AST** | 25.17 ± 12.83 | 25.54 ± 11.56 | 24.99 ± 13.96 | 25.64 ± 14.63 | 24.53 ± 10.74 | **0.039** |
| **HbA1c** | 6.05 ± 1.18 | 5.95 ± 1.05 | 5.99 ± 1.07 | 6.06 ± 1.22 | 6.18 ± 1.34 | **<.001** |
| **GLU** | 6.20 ± 2.60 | 6.13 ± 2.39 | 6.17 ± 2.55 | 6.19 ± 2.60 | 6.33 ± 2.84 | 0.123 |
| **ALB** | 41.42 ± 3.38 | 41.11 ± 3.43 | 41.43 ± 3.41 | 41.61 ± 3.29 | 41.55 ± 3.38 | **<.001** |
| **Sex** |  |  |  |  |  | **<.001** |
| **Female** | 3490 (50.73) | 650 (37.79) | 798 (46.40) | 940 (54.65) | 1102 (64.07) |  |
| **Male** | 3390 (49.27) | 1070 (62.21) | 922 (53.60) | 780 (45.35) | 618 (35.93) |  |
| **Race** |  |  |  |  |  | **<.001** |
| **Hispanics** | 1040 (15.12) | 200 (11.63) | 252 (14.65) | 290 (16.86) | 298 (17.33) |  |
| **Non-Hispanics White** | 3571 (51.9) | 1150 (66.86) | 993 (57.73) | 824 (47.91) | 604 (35.12) |  |
| **Non-Hispanics Black** | 1920 (27.91) | 306 (17.79) | 394 (22.91) | 512 (29.77) | 708 (41.16) |  |
| **Other** | 349 (5.07) | 64 (3.72) | 81 (4.71) | 94 (5.47) | 110 (6.40) |  |
| **Education level** |  |  |  |  |  | **0.018** |
| **≤ High school** | 3862 (56.13) | 949 (55.17) | 972 (56.51) | 1002 (58.26) | 939 (54.59) |  |
| **College** | 1766 (25.67) | 426 (24.77) | 421 (24.48) | 440 (25.58) | 479 (27.85) |  |
| **> College** | 1252 (18.2) | 345 (20.06) | 327 (19.01) | 278 (16.16) | 302 (17.56) |  |
| **Marital status** |  |  |  |  |  | 0.265 |
| **Not married** | 3202 (46.54) | 787 (45.76) | 787 (45.76) | 792 (46.05) | 836 (48.60) |  |
| **Married or living with partner** | 3678 (53.46) | 933 (54.24) | 933 (54.24) | 928 (53.95) | 884 (51.40) |  |
| **Family income-poverty ratio** |  |  |  |  |  | **<.001** |
| **<1.3** | 1924 (27.97) | 439 (25.52) | 446 (25.93) | 497 (28.90) | 542 (31.51) |  |
| **1.3-3.5** | 3291 (47.83) | 875 (50.87) | 842 (48.95) | 807 (46.92) | 767 (44.59) |  |
| **>3.5** | 1665 (24.2) | 406 (23.60) | 432 (25.12) | 416 (24.19) | 411 (23.90) |  |
| **Hypertension** |  |  |  |  |  | **<.001** |
| **No** | 2398 (34.85) | 552 (32.09) | 566 (32.91) | 625 (36.34) | 655 (38.08) |  |
| **Yes** | 4482 (65.15) | 1168 (67.91) | 1154 (67.09) | 1095 (63.66) | 1065 (61.92) |  |
| **Hyperlipidemia** |  |  |  |  |  | 0.140 |
| **No** | 3554 (51.66) | 915 (53.20) | 873 (50.76) | 857 (49.83) | 909 (52.85) |  |
| **Yes** | 3326 (48.34) | 805 (46.80) | 847 (49.24) | 863 (50.17) | 811 (47.15) |  |
| **Diabetes mellitus** |  |  |  |  |  | 0.806 |
| **No** | 4957 (72.05) | 1255 (72.97) | 1237 (71.92) | 1249 (72.62) | 1216 (70.70) |  |
| **Yes** | 1736 (25.23) | 422 (24.53) | 438 (25.47) | 424 (24.65) | 452 (26.28) |  |
| **Borderline** | 187 (2.72) | 43 (2.50) | 45 (2.62) | 47 (2.73) | 52 (3.02) |  |
| **CHF** |  |  |  |  |  | **<.001** |
| **No** | 6187 (89.93) | 1451 (84.36) | 1531 (89.01) | 1593 (92.62) | 1612 (93.72) |  |
| **Yes** | 693 (10.07) | 269 (15.64) | 189 (10.99) | 127 (7.38) | 108 (6.28) |  |
| **Smoking status** |  |  |  |  |  | **<.001** |
| **Never** | 3418 (49.68) | 777 (45.17) | 857 (49.83) | 897 (52.15) | 887 (51.57) |  |
| **Now** | 944 (13.72) | 176 (10.23) | 197 (11.45) | 249 (14.48) | 322 (18.72) |  |
| **Ever** | 2518 (36.6) | 767 (44.59) | 666 (38.72) | 574 (33.37) | 511 (29.71) |  |
| **Alcohol Use** |  |  |  |  |  | **0.033** |
| **Never** | 1892 (27.64) | 519 (30.32) | 463 (27.11) | 464 (27.12) | 446 (26.02) |  |
| **Now** | 3279 (47.9) | 820 (47.90) | 817 (47.83) | 806 (47.11) | 836 (48.77) |  |
| **Ever** | 1674 (24.46) | 373 (21.79) | 428 (25.06) | 441 (25.77) | 432 (25.20) |  |

Note: Continuous variables are reported as the mean value with the standard deviation (SD) and categorical variables are reported as the frequency with the percentage in parentheses. ^a^ Bold value means statistically significant

Abbreviation: CKD: chronic kidney disease; LMR: lymphocyte-to-monocyte ratio; BMI: body mass index; eGFR: estimated glomerular filtration rate; BUN: blood urea nitrogen; ALT: alanine transaminase; AST: aspartate transaminase; HbA1c: glycosylated hemoglobin; ALB: albumin; CHF: congestive heart failure.

**Table S5.** The Cox regression analysis evaluates the association between the SII index with the all-cause mortality of the CKD population.

| **Variables** | **Subgroup** | **Hazard ratio (95%CI)** | **P** |
| --- | --- | --- | --- |
| **Model 1** |  |  |  |
| **Group** | Q1 | Reference |  |
|  | Q2 | 1.17(1.04-1.32) | **0.010** |
|  | Q3 | 1.30(1.16-1.46) | **<0.001** |
|  | Q4 | 1.66(1.48-1.85) | **<0.001** |
| **Model 2** |  |  |  |
| **Group** | Q1 | Reference |  |
|  | Q2 | 1.17(1.03-1.32) | **0.012** |
|  | Q3 | 1.23(1.09-1.38) | **0.001** |
|  | Q4 | 1.57(1.40-1.76) | **<0.001** |
| **Age** | / | 1.09(1.08-1.09) | **<0.001** |
| **Sex** | Female | Reference |  |
|  | Male | 1.40(1.29-1.51) | **<0.001** |
| **Race** | Hispanics | Reference |  |
|  | Non-Hispanics White | 1.16(1.02-1.31) | **0.025** |
|  | Non-Hispanics Black | 1.26(1.09-1.45) | **0.002** |
|  | Other | 0.97(0.74-1.26) | 0.790 |
| **Model 3** |  |  |  |
| **Group** | Q1 | Reference |  |
|  | Q2 | 1.13(0.99-1.27) | 0.056 |
|  | Q3 | 1.13(1.01-1.28) | **0.039** |
|  | Q4 | 1.39(1.24-1.57) | **<0.001** |
| **Sex** | Female | Reference |  |
|  | Male | 1.51(1.38-1.64) | **<0.001** |
| **Race** | Hispanics | Reference |  |
|  | Non-Hispanics White | 1.44(1.27-1.65) | **<0.001** |
|  | Non-Hispanics Black | 1.22(1.05-1.41) | **0.009** |
|  | Other | 0.96(0.74-1.26) | 0.788 |
| **Marital status** | Not married | Reference |  |
|  | Married or living with partner | 0.79(0.72-0.86) | **<0.001** |
| **Education level** | ≤ High school | Reference |  |
|  | College | 0.94(0.85-1.04) | 0.248 |
|  | >College | 0.84(0.74-0.96) | **0.007** |
| **Family income-poverty ratio** | <1.3 | Reference |  |
|  | 1.3-3.5 | 0.84(0.77-0.92) | **<0.001** |
|  | >3.5 | 0.68(0.60-0.78) | **<0.001** |
| **Smoking status** | Never | Reference |  |
|  | Now | 1.56(1.36-1.79) | **<0.001** |
|  | Ever | 1.17(1.08-1.28) | **<0.001** |
| **Hypertension** | No | Reference |  |
|  | Yes | 1.04(0.96-1.14) | 0.334 |
| **Hyperlipidemia** | No | Reference |  |
|  | Yes | 0.88(0.81-0.95) | **0.001** |
| **Diabetes mellitus** | No | Reference |  |
|  | Yes | 1.27(1.14-1.42) | **<0.001** |
|  | Borderline | 1.17(0.91-1.49) | 0.214 |
| **CHF** | No | Reference |  |
|  | Yes | 1.64(1.47-1.83) | **<0.001** |
| **Age** | / | 1.08(1.07-1.09) | **<0.001** |
| **BMI** | / | 0.99(0.99-1.00) | 0.088 |
| **eGFR** | / | 1.00(0.99-1.00) | 0.323 |
| **BUN** | / | 1.07(1.06-1.09) | **<0.001** |
| **ALT** | / | 0.99(0.99-0.99) | **0.049** |
| **AST** | / | 1.01(1.00-1.01) | **0.001** |
| **HbA1c** | / | 1.04(0.99-1.09) | 0.087 |
| **ALB** | / | 0.94(0.93-0.95) | **<0.001** |

**Note: (1)** Model 1: univariate Cox analysis; Model 2: adjustments for age, sex, and race; Model 3: accounted for age, sex, race, marital status, educational level, family income-poverty ratio, smoking status, hypertension, hyperlipidemia, diabetes mellitus, CHF, BMI, eGFR, BUN, ALT, AST, HbA1c, and ALB. (2) Bold value means statically significant.

Abbreviation: CKD: chronic kidney disease; SII: systemic immune-inflammation index; CI: Confidence interval; Q: quartile; CHF: Congestive heart failure; BMI: body mass index; eGFR: estimated glomerular filtration rate; BUN: blood urea nitrogen; ALT: alanine transaminase; AST: aspartate transaminase; HbA1c: glycosylated hemoglobin; ALB: albumin.

**Table S6.** The Cox regression analysis evaluates the association between the NLR index with the all-cause mortality of the CKD population.

| **Variables** | **Subgroup** | **Hazard ratio (95%CI)** | **P** |
| --- | --- | --- | --- |
| **Model 1** |  |  |  |
| **Group** | Q1 | Reference |  |
|  | Q2 | 1.19(1.05-1.35) | **0.006** |
|  | Q3 | 1.66(1.48-1.87) | **<0.001** |
|  | Q4 | 2.41(2.15-2.70) | **<0.001** |
| **Model 2** |  |  |  |
| **Group** | Q1 | Reference |  |
|  | Q2 | 1.10(0.97-1.25) | 0.138 |
|  | Q3 | 1.36(1.20-1.53) | **<0.001** |
|  | Q4 | 1.80(1.60-2.02) | **<0.001** |
| **Age** | / | 1.08(1.07-1.09) | **<0.001** |
| **Sex** | Female | Reference |  |
|  | Male | 1.32(1.22-1.42) | **<0.001** |
| **Race** | Hispanics | Reference |  |
|  | Non-Hispanics White | 1.13(0.99-1.28) | 0.059 |
|  | Non-Hispanics Black | 1.27(1.10-1.47) | **0.001** |
|  | Other | 0.94(0.72-1.23) | 0.663 |
| **Model 3** |  |  |  |
| **Group** | Q1 | Reference |  |
|  | Q2 | 1.06(0.93-1.20) | 0.379 |
|  | Q3 | 1.21(1.07-1.36) | **0.003** |
|  | Q4 | 1.52(1.35-1.72) | **<0.001** |
| **Sex** | Female | Reference |  |
|  | Male | 1.45(1.33-1.58) | **<0.001** |
| **Race** | Hispanics | Reference |  |
|  | Non-Hispanics White | 1.42(1.25-1.62) | **<0.001** |
|  | Non-Hispanics Black | 1.23(1.06-1.43) | **0.007** |
|  | Other | 0.95(0.73-1.25) | 0.725 |
| **Marital status** | Not married | Reference |  |
|  | Married or living with partner | 0.79(0.73-0.87) | **<0.001** |
| **Education level** | ≤ High school | Reference |  |
|  | College | 0.94(0.85-1.04) | 0.231 |
|  | >College | 0.83(0.73-0.94) | **0.003** |
| **Family income-poverty ratio** | <1.3 | Reference |  |
|  | 1.3-3.5 | 0.85(0.78-0.93) | **0.001** |
|  | >3.5 | 0.68(0.60-0.78) | **<0.001** |
| **Smoking status** | Never | Reference |  |
|  | Now | 1.57(1.37-1.80) | **<0.001** |
|  | Ever | 1.17(1.08-1.28) | **<0.001** |
| **Hypertension** | No | Reference |  |
|  | Yes | 1.04(0.96-1.14) | 0.352 |
| **Hyperlipidemia** | No | Reference |  |
|  | Yes | 0.88(0.81-0.95) | **0.002** |
| **Diabetes mellitus** | No | Reference |  |
|  | Yes | 1.27(1.13-1.42) | **<0.001** |
|  | Borderline | 1.15(0.90-1.47) | 0.261 |
| **CHF** | No | Reference |  |
|  | Yes | 1.61(1.44-1.80) | **<0.001** |
| **Age** | / | 1.08(1.07-1.08) | **<0.001** |
| **BMI** | / | 0.99(0.99-1.00) | 0.129 |
| **eGFR** | / | 1.00(0.99-1.00) | 0.372 |
| **BUN** | / | 1.07(1.05-1.08) | **<0.001** |
| **ALT** | / | 0.99(0.99-0.99) | **0.039** |
| **AST** | / | 1.01(1.00-1.01) | **<0.001** |
| **HbA1c** | / | 1.04(0.99-1.09) | 0.065 |
| **ALB** | / | 0.94(0.93-0.95) | **<0.001** |

Note: (1) Model 1: univariate Cox analysis; Model 2: adjustments for age, sex, and race; Model 3: accounted for age, sex, race, marital status, educational level, family income-poverty ratio, smoking status, hypertension, hyperlipidemia, diabetes mellitus, CHF, BMI, eGFR, BUN, ALT, AST, HbA1c, and ALB. (2) Bold value means statically significant.

Abbreviation: CKD: chronic kidney disease; NLR: neutrophil-to-lymphocyte ratio; CI: Confidence interval; Q: quartile; CHF: Congestive heart failure; BMI: body mass index; eGFR: estimated glomerular filtration rate; BUN: blood urea nitrogen; ALT: alanine transaminase; AST: aspartate transaminase; HbA1c: glycosylated hemoglobin; ALB: albumin.

**Table S7.** The Cox regression analysis evaluates the association between the PLR index with the all-cause mortality of the CKD population.

| **Variables** | **Subgroup** | **Hazard ratio (95%CI)** | **P** |
| --- | --- | --- | --- |
| **Model 1** |  |  |  |
| **Group** | Q1 | Reference |  |
|  | Q2 | 0.95(0.85-1.07) | 0.415 |
|  | Q3 | 0.93(0.83-1.04) | 0.200 |
|  | Q4 | 1.32(1.18-1.47) | **<0.001** |
| **Model 2** |  |  |  |
| **Group** | Q1 | Reference |  |
|  | Q2 | 0.94(0.84-1.05) | 0.285 |
|  | Q3 | 0.91(0.81-1.02) | 0.113 |
|  | Q4 | 1.19(1.07-1.32) | **0.002** |
| **Age** | / | 1.09(1.08-1.09) | **<0.001** |
| **Sex** | Female | Reference |  |
|  | Male | 1.41(1.30-1.52) | **<0.001** |
| **Race** | Hispanics | Reference |  |
|  | Non-Hispanics White | 1.18(1.04-1.34) | **0.010** |
|  | Non-Hispanics Black | 1.19(1.03-1.37) | **0.016** |
|  | Other | 0.96(0.74-1.26) | 0.788 |
| **Model 3** |  |  |  |
| **Group** | Q1 | Reference |  |
|  | Q2 | 0.97(0.87-1.09) | 0.641 |
|  | Q3 | 0.99(0.88-1.11) | 0.815 |
|  | Q4 | 1.22(1.10-1.37) | **<0.001** |
| **Sex** | Female | Reference |  |
|  | Male | 1.51(1.39-1.65) | **<0.001** |
| **Race** | Hispanics | Reference |  |
|  | Non-Hispanics White | 1.47(1.29-1.67) | **<0.001** |
|  | Non-Hispanics Black | 1.18(1.01-1.36) | **0.032** |
|  | Other | 0.97(0.75-1.27) | 0.840 |
| **Marital status** | Not married | Reference |  |
|  | Married or living with partner | 0.79(0.72-0.86) | **<0.001** |
| **Education level** | ≤ High school | Reference |  |
|  | College | 0.95(0.86-1.05) | 0.271 |
|  | >College | 0.84(0.74-0.96) | **0.008** |
| **Family income-poverty ratio** | <1.3 | Reference |  |
|  | 1.3-3.5 | 0.84(0.77-0.93) | **<0.001** |
|  | >3.5 | 0.69(0.60-0.78) | **<0.001** |
| **Smoking status** | Never | Reference |  |
|  | Now | 1.62(1.42-1.86) | **<0.001** |
|  | Ever | 1.19(1.09-1.29) | **<0.001** |
| **Hypertension** | No | Reference |  |
|  | Yes | 1.05(0.96-1.15) | 0.294 |
| **Hyperlipidemia** | No | Reference |  |
|  | Yes | 0.88(0.81-0.95) | **0.001** |
| **Diabetes mellitus** | No | Reference |  |
|  | Yes | 1.27(1.14-1.42) | **<0.001** |
|  | Borderline | 1.17(0.92-1.50) | 0.212 |
| **CHF** | No | Reference |  |
|  | Yes | 1.65(1.48-1.84) | **<0.001** |
| **Age** | / | 1.08(1.07-1.09) | **<0.001** |
| **BMI** | / | 1.00(0.99-1.00) | 0.163 |
| **eGFR** | / | 1.00(1.00-1.00) | 0.359 |
| **BUN** | / | 1.07(1.06-1.09) | **<0.001** |
| **ALT** | / | 1.00(0.99-1.00) | 0.061 |
| **AST** | / | 1.01(1.00-1.01) | **0.001** |
| **HbA1c** | / | 1.05(1.00-1.09) | **0.042** |
| **ALB** | / | 0.94(0.93-0.95) | **<0.001** |

Note: (1) Model 1: univariate Cox analysis; Model 2: adjustments for age, sex, and race; Model 3: accounted for age, sex, race, marital status, educational level, family income-poverty ratio, smoking status, hypertension, hyperlipidemia, diabetes mellitus, CHF, BMI, eGFR, BUN, ALT, AST, HbA1c, and ALB. (2) Bold value means statically significant.

Abbreviation: CKD: chronic kidney disease; PLR: platelet-to-lymphocyte ratio; CI: Confidence interval; Q: quartile; CHF: Congestive heart failure; BMI: body mass index; eGFR: estimated glomerular filtration rate; BUN: blood urea nitrogen; ALT: alanine transaminase; AST: aspartate transaminase; HbA1c: glycosylated hemoglobin; ALB: albumin.

**Table S8.** The Cox regression analysis evaluates the association between the LMR index with the all-cause mortality of the CKD population.

| **Variables** | **Subgroup** | **Hazard ratio (95%CI)** | **P** |
| --- | --- | --- | --- |
| **Model 1** |  |  |  |
| **Group** | Q1 | Reference |  |
|  | Q2 | 0.66(0.59-0.72) | **<0.001** |
|  | Q3 | 0.44(0.40-0.49) | **<0.001** |
|  | Q4 | 0.36(0.32-0.40) | **<0.001** |
| **Model 2** |  |  |  |
| **Group** | Q1 | Reference |  |
|  | Q2 | 0.77(0.70-0.85) | **<0.001** |
|  | Q3 | 0.64(0.57-0.71) | **<0.001** |
|  | Q4 | 0.65(0.57-0.73) | **<0.001** |
| **Age** | / | 1.08(1.08-1.09) | **<0.001** |
| **Sex** | Female | Reference |  |
|  | Male | 1.28(1.18-1.38) | **<0.001** |
| **Race** | Hispanics | Reference |  |
|  | Non-Hispanics White | 1.12(0.99-1.28) | 0.070 |
|  | Non-Hispanics Black | 1.21(1.05-1.40) | **0.008** |
|  | Other | 0.97(0.75-1.27) | 0.840 |
| **Model 3** |  |  |  |
| **Group** | Q1 | Reference |  |
|  | Q2 | 0.83(0.75-0.92) | **<0.001** |
|  | Q3 | 0.73(0.65-0.82) | **<0.001** |
|  | Q4 | 0.74(0.65-0.83) | **<0.001** |
| **Sex** | Female | Reference |  |
|  | Male | 1.42(1.30-1.55) | **<0.001** |
| **Race** | Hispanics | Reference |  |
|  | Non-Hispanics White | 1.43(1.25-1.63) | **<0.001** |
|  | Non-Hispanics Black | 1.19(1.03-1.38) | **0.019** |
|  | Other | 0.99(0.75-1.29) | 0.911 |
| **Marital status** | Not married | Reference |  |
|  | Married or living with partner | 0.79(0.73-0.87) | **<0.001** |
| **Education level** | ≤ High school | Reference |  |
|  | College | 0.95(0.86-1.05) | 0.282 |
|  | >College | 0.84(0.74-0.95) | **0.005** |
| **Family income-poverty ratio** | <1.3 | Reference |  |
|  | 1.3-3.5 | 0.85(0.77-0.93) | **0.001** |
|  | >3.5 | 0.69(0.60-0.78) | **<0.001** |
| **Smoking status** | Never | Reference |  |
|  | Now | 1.63(1.42-1.87) | **<0.001** |
|  | Ever | 1.18(1.08-1.28) | **<0.001** |
| **Hypertension** | No | Reference |  |
|  | Yes | 1.04(0.95-1.13) | 0.438 |
| **Hyperlipidemia** | No | Reference |  |
|  | Yes | 0.89(0.82-0.96) | **0.003** |
| **Diabetes mellitus** | No | Reference |  |
|  | Yes | 1.27(1.14-1.42) | **<0.001** |
|  | Borderline | 1.18(0.93-1.51) | 0.177 |
| **CHF** | No | Reference |  |
|  | Yes | 1.63(1.46-1.82) | **<0.001** |
| **Age** | / | 1.08(1.07-1.08) | **<0.001** |
| **BMI** | / | 1.00(0.99-1.00) | 0.231 |
| **eGFR** | / | 1.00(1.00-1.00) | 0.365 |
| **BUN** | / | 1.07(1.05-1.08) | **<0.001** |
| **ALT** | / | 1.00(0.99-1.00) | 0.103 |
| **AST** | / | 1.01(1.00-1.01) | **0.006** |
| **HbA1c** | / | 1.04(0.99-1.09) | 0.063 |
| **ALB** | / | 0.94(0.93-0.95) | **<0.001** |

Note: (1) Model 1: univariate Cox analysis; Model 2: adjustments for age, sex, and race; Model 3: accounted for age, sex, race, marital status, educational level, family income-poverty ratio, smoking status, hypertension, hyperlipidemia, diabetes mellitus, CHF, BMI, eGFR, BUN, ALT, AST, HbA1c, and ALB. (2) Bold value means statically significant.

Abbreviation: CKD: chronic kidney disease; LMR: lymphocyte-to-monocyte ratio; CI: Confidence interval; Q: quartile; CHF: Congestive heart failure; BMI: body mass index; eGFR: estimated glomerular filtration rate; BUN: blood urea nitrogen; ALT: alanine transaminase; AST: aspartate transaminase; HbA1c: glycosylated hemoglobin; ALB: albumin.

**Table S9**. The C-index and AUC of the predictive value of each inflammation index in the survival of the CKD population.

| **Inflammation index** | **AUC of ROC (95%CI)** | **C-index (95%CI)** | **C-index (95%CI)** |
| --- | --- | --- | --- |
| **SII** | 0.584 (0.571 – 0.597) | 0.563 (0.551 – 0.575) | 0.779 (0.769 – 0.789) |
| **NLR** | 0.613 (0.599 – 0.626) | 0.612 (0.600 – 0.624) | 0.785 (0.775 – 0.794) |
| **PLR** | 0.567 (0.554 – 0.581) | 0.537 (0.525 – 0.549) | 0.768 (0.758 – 0.779) |
| **LMR** | 0.621 (0.607 – 0.634) | 0.624 (0.612 – 0.636) | 0.789 (0.781 – 0.797) |

^a^ the C-index for a single indicator; ^b^ the C-index for systemic inflammatory indicator combined with other clinical characteristics.

Abbreviation: C-index: Concordance index; AUC: Area under the curve; CKD: chronic kidney disease; ROC: Receiver operating characteristic; CI: Confidence interval; SII: systemic immune-inflammation index; NLR: neutrophil-to-lymphocyte ratio; PLR: platelet-to-lymphocyte ratio; LMR: lymphocyte-to-monocyte ratio.

**Table S10.** Subgroup analysis for evaluating the systemic inflammatory indicators with the survival of the CKD population with stage III.

| **Variables** | **Hazard ratio (95%CI)** | **P** |
| --- | --- | --- |
| **SII** |  |  |
| **Q1** | Reference |  |
| **Q2** | 1.02(0.89-1.17) | 0.733 |
| **Q3** | 1.05(0.91-1.20) | 0.512 |
| **Q4** | 1.33(1.17-1.52) | **<0.001** |
| **NLR** |  |  |
| **Q1** | Reference |  |
| **Q2** | 1.10(0.96-1.27) | 0.185 |
| **Q3** | 1.22(1.06-1.40) | **0.006** |
| **Q4** | 1.54(1.34-1.77) | **<0.001** |
| **PLR** |  |  |
| **Q1** | Reference |  |
| **Q2** | 0.94(0.83-1.08) | 0.378 |
| **Q3** | 0.97(0.85-1.11) | 0.663 |
| **Q4** | 1.19(1.05-1.34) | **0.008** |
| **LMR** |  |  |
| **Q1** | Reference |  |
| **Q2** | 0.85(0.76-0.96) | **0.006** |
| **Q3** | 0.74(0.65-0.84) | **<0.001** |
| **Q4** | 0.74(0.64-0.85) | **<0.001** |

Note: the results were accounted for age, sex, race, marital status, educational level, family income-poverty ratio, smoking status, hypertension, hyperlipidemia, diabetes mellitus, CHF, BMI, eGFR, BUN, ALT, AST, HbA1c, and ALB. (2) Bold value means statically significant.

Abbreviation: CKD: chronic kidney disease; CI: confidence interval; SII: systemic immune-inflammation index; NLR: neutrophil-to-lymphocyte ratio; PLR: platelet-to-lymphocyte ratio; LMR: lymphocyte-to-monocyte ratio; CHF: Congestive heart failure; BMI: body mass index; eGFR: estimated glomerular filtration rate; BUN: blood urea nitrogen; ALT: alanine transaminase; AST: aspartate transaminase; HbA1c: glycosylated hemoglobin; ALB: albumin.

**Table S11.** Subgroup analysis for evaluating the systemic inflammatory indicators with the survival of the CKD population with stage IV.

| **Variables** | **Hazard ratio (95%CI)** | **P** |
| --- | --- | --- |
| **SII** |  |  |
| **Q1** | Reference |  |
| **Q2** | 1.51(1.08-2.12) | **0.015** |
| **Q3** | 1.43(1.01-2.01) | **0.041** |
| **Q4** | 1.61(1.13-2.30) | **0.009** |
| **NLR** |  |  |
| **Q1** | Reference |  |
| **Q2** | 0.70(0.47-1.03) | 0.070 |
| **Q3** | 0.93(0.65-1.34) | 0.698 |
| **Q4** | 1.01(0.71-1.44) | 0.960 |
| **PLR** |  |  |
| **Q1** | Reference |  |
| **Q2** | 1.36(0.98-1.90) | 0.069 |
| **Q3** | 1.12(0.80-1.57) | 0.526 |
| **Q4** | 1.45(1.05-1.98) | **0.022** |
| **LMR** |  |  |
| **Q1** | Reference |  |
| **Q2** | 1.06(0.80-1.41) | 0.686 |
| **Q3** | 0.80(0.55-1.16) | 0.231 |
| **Q4** | 0.66(0.48-0.92) | **0.014** |

Note: the results were accounted for age, sex, race, marital status, educational level, family income-poverty ratio, smoking status, hypertension, hyperlipidemia, diabetes mellitus, CHF, BMI, eGFR, BUN, ALT, AST, HbA1c, and ALB. (2) Bold value means statically significant.

Abbreviation: CKD: chronic kidney disease; CI: confidence interval; SII: systemic immune-inflammation index; NLR: neutrophil-to-lymphocyte ratio; PLR: platelet-to-lymphocyte ratio; LMR: lymphocyte-to-monocyte ratio; CHF: Congestive heart failure; BMI: body mass index; eGFR: estimated glomerular filtration rate; BUN: blood urea nitrogen; ALT: alanine transaminase; AST: aspartate transaminase; HbA1c: glycosylated hemoglobin; ALB: albumin.

**Table S12.** Subgroup analysis for evaluating the systemic inflammatory indicators with the survival of the CKD population with stage V.

| **Variables** | **Hazard ratio (95%CI)** | **P** |
| --- | --- | --- |
| **SII** |  |  |
| **Q1** | Reference |  |
| **Q2** | 1.94(0.95-3.97) | 0.071 |
| **Q3** | 1.97(1.01-3.84) | **0.046** |
| **Q4** | 1.86(0.97-3.58) | 0.063 |
| **NLR** |  |  |
| **Q1** | Reference |  |
| **Q2** | 0.72(0.31-1.71) | 0.458 |
| **Q3** | 1.42(0.68-2.96) | 0.348 |
| **Q4** | 1.28(0.63-2.58) | 0.490 |
| **PLR** |  |  |
| **Q1** | Reference |  |
| **Q2** | 0.62(0.30-1.25) | 0.177 |
| **Q3** | 1.17(0.60-2.27) | 0.643 |
| **Q4** | 1.32(0.75-2.30) | 0.332 |
| **LMR** |  |  |
| **Q1** | Reference |  |
| **Q2** | 0.64(0.38-1.07) | 0.089 |
| **Q3** | 0.75(0.42-1.34) | 0.327 |
| **Q4** | 0.84(0.43-1.65) | 0.615 |

Note: the results were accounted for age, sex, race, marital status, educational level, family income-poverty ratio, smoking status, hypertension, hyperlipidemia, diabetes mellitus, CHF, BMI, eGFR, BUN, ALT, AST, HbA1c, and ALB. (2) Bold value means statically significant.

Abbreviation: CKD: chronic kidney disease; CI: confidence interval; SII: systemic immune-inflammation index; NLR: neutrophil-to-lymphocyte ratio; PLR: platelet-to-lymphocyte ratio; LMR: lymphocyte-to-monocyte ratio; CHF: Congestive heart failure; BMI: body mass index; eGFR: estimated glomerular filtration rate; BUN: blood urea nitrogen; ALT: alanine transaminase; AST: aspartate transaminase; HbA1c: glycosylated hemoglobin; ALB: albumin.

**Table S13.** Sensitive analysis in evaluating the association between SII with the all-cause mortality of the CKD population

| **SII** | **Model 1** |  | **Model 2** |  | **Model 3** |  |
| --- | --- | --- | --- | --- | --- | --- |
|  | **HR (95% CI)** | **P** | **HR (95% CI)** | **P** | **HR (95% CI)** | **P** |
| **Q1** | Reference |  | Reference |  | Reference |  |
| **Q2** | 1.18(1.04-1.34) | **0.009** | 1.20(1.06-1.36) | **0.005** | 1.19(1.04-1.35) | **0.009** |
| **Q3** | 1.30(1.15-1.47) | **<0.001** | 1.23(1.08-1.39) | **0.002** | 1.15(1.02-1.31) | **0.027** |
| **Q4** | 1.72(1.53-1.94) | **<0.001** | 1.59(1.41-1.80) | **<0.001** | 1.43(1.26-1.61) | **<0.001** |

Note: (1) Model 1: univariate Cox analysis; Model 2: adjustments for age, sex, and race; Model 3: accounted for age, sex, race, marital status, educational level, family income-poverty ratio, smoking status, hypertension, hyperlipidemia, diabetes mellitus, CHF, BMI, eGFR, BUN, ALT, AST, HbA1c, ALB, and dialysis. (2) Bold value means statically significant.

Abbreviation: CKD chronic kidney disease; SII: systemic immune-inflammation index; HR: hazard ratio; CI: confidence interval; CHF: Congestive heart failure; BMI: body mass index; eGFR: estimated glomerular filtration rate; BUN: blood urea nitrogen; ALT: alanine transaminase; AST: aspartate transaminase; HbA1c: glycosylated hemoglobin; ALB: albumin.

**Table S14.** Sensitive analysis in evaluating the association between NLR with the all-cause mortality of the CKD population

| **NLR** | **Model 1** |  | **Model 2** |  | **Model 3** |  |
| --- | --- | --- | --- | --- | --- | --- |
|  | **HR (95% CI)** | **P** | **HR (95% CI)** | **P** | **HR (95% CI)** | **P** |
| **Q1** | Reference |  | Reference |  | Reference |  |
| **Q2** | 1.25(1.09-1.43) | **0.001** | 1.17(1.02-1.33) | **0.025** | 1.13(0.99-1.29) | 0.079 |
| **Q3** | 1.72(1.52-1.95) | **<0.001** | 1.40(1.23-1.59) | **<0.001** | 1.26(1.10-1.43) | **0.001** |
| **Q4** | 2.55(2.26-2.88) | **<0.001** | 1.84(1.62-2.09) | **<0.001** | 1.57(1.38-1.79) | **<0.001** |

Note: (1) Model 1: univariate Cox analysis; Model 2: adjustments for age, sex, and race; Model 3: accounted for age, sex, race, marital status, educational level, family income-poverty ratio, smoking status, hypertension, hyperlipidemia, diabetes mellitus, CHF, BMI, eGFR, BUN, ALT, AST, HbA1c, ALB, and dialysis. (2) Bold value means statically significant.

Abbreviation: CKD chronic kidney disease; NLR: neutrophil-to-lymphocyte ratio; HR: hazard ratio; CI: confidence interval; CHF: Congestive heart failure; BMI: body mass index; eGFR: estimated glomerular filtration rate; BUN: blood urea nitrogen; ALT: alanine transaminase; AST: aspartate transaminase; HbA1c: glycosylated hemoglobin; ALB: albumin.

**Table S15.** Sensitive analysis in evaluating the association between PLR with the all-cause mortality of the CKD population

| **PLR** | **Model 1** |  | **Model 2** |  | **Model 3** |  |
| --- | --- | --- | --- | --- | --- | --- |
|  | **HR (95% CI)** | **P** | **HR (95% CI)** | **P** | **HR (95% CI)** | **P** |
| **Q1** | Reference |  | Reference |  | Reference |  |
| **Q2** | 0.96(0.85-1.09) | 0.546 | 0.96(0.85-1.09) | 0.561 | 1.02(0.90-1.16) | 0.731 |
| **Q3** | 0.92(0.81-1.04) | 0.178 | 0.89(0.79-1.01) | 0.071 | 0.97(0.86-1.10) | 0.615 |
| **Q4** | 1.33(1.19-1.49) | **<0.001** | 1.20(1.07-1.34) | **0.002** | 1.22(1.09-1.37) | **0.001** |

Note: (1) Model 1: univariate Cox analysis; Model 2: adjustments for age, sex, and race; Model 3: accounted for age, sex, race, marital status, educational level, family income-poverty ratio, smoking status, hypertension, hyperlipidemia, diabetes mellitus, CHF, BMI, eGFR, BUN, ALT, AST, HbA1c, ALB, and dialysis. (2) Bold value means statically significant.

Abbreviation: CKD chronic kidney disease; PLR: platelet-to-lymphocyte ratio; HR: hazard ratio; CI: confidence interval; CHF: Congestive heart failure; BMI: body mass index; eGFR: estimated glomerular filtration rate; BUN: blood urea nitrogen; ALT: alanine transaminase; AST: aspartate transaminase; HbA1c: glycosylated hemoglobin; ALB: albumin.

**Table S16.** Sensitive analysis in evaluating the association between LMR with the all-cause mortality of the CKD population

| **LMR** | **Model 1** |  | **Model 2** |  | **Model 3** |  |
| --- | --- | --- | --- | --- | --- | --- |
|  | **HR (95% CI)** | **P** | **HR (95% CI)** | **P** | **HR (95% CI)** | **P** |
| **Q1** | Reference |  | Reference |  | Reference |  |
| **Q2** | 0.66(0.59-0.73) | **<0.001** | 0.79(0.71-0.88) | **<0.001** | 0.86(0.77-0.95) | **0.004** |
| **Q3** | 0.44(0.39-0.49) | **<0.001** | 0.63(0.56-0.71) | **<0.001** | 0.73(0.65-0.82) | **<0.001** |
| **Q4** | 0.35(0.31-0.39) | **<0.001** | 0.65(0.57-0.74) | **<0.001** | 0.72(0.63-0.82) | **<0.001** |

Note: (1) Model 1: univariate Cox analysis; Model 2: adjustments for age, sex, and race; Model 3: accounted for age, sex, race, marital status, educational level, family income-poverty ratio, smoking status, hypertension, hyperlipidemia, diabetes mellitus, CHF, BMI, eGFR, BUN, ALT, AST, HbA1c, ALB, and dialysis. (2) Bold value means statically significant.

Abbreviation: CKD chronic kidney disease; LMR: lymphocyte-to-monocyte ratio; HR: hazard ratio; CI: confidence interval; CHF: Congestive heart failure; BMI: body mass index; eGFR: estimated glomerular filtration rate; BUN: blood urea nitrogen; ALT: alanine transaminase; AST: aspartate transaminase; HbA1c: glycosylated hemoglobin; ALB: albumin.
